# Differentiating factors of intra-articular injectables have a meaningful impact on knee osteoarthritis outcomes: a network meta-analysis

**DOI:** 10.1007/s00167-019-05763-1

**Published:** 2020-01-03

**Authors:** Mark Phillips, Christopher Vannabouathong, Tahira Devji, Rahil Patel, Zoya Gomes, Ashaka Patel, Mykaelah Dixon, Mohit Bhandari

**Affiliations:** 1 Department of Health Research Methods, Evidence, and Impact, McMaster University, Hamilton, ON Canada; 2 Division of Orthopaedic Surgery, Centre for Evidence-Based Orthopaedics, McMaster University, Hamilton, ON Canada; 3 OrthoEvidence Inc., Burlington, ON Canada; 4 Faculty of Health Sciences, McMaster University, Hamilton, ON Canada

## Abstract

**Purpose:**

There are a number of developments in intra-articular therapies that have been determined to be differentiating factors within the classes of treatments. This study evaluated the efficacy and safety of intra-articular treatments of primary knee osteoarthritis in the short term (3 months follow-up), using a network meta-analysis design, while taking within-class differentiating factors into consideration.

**Methods:**

A literature search of MEDLINE (through OVID), EMBASE (through OVID), Cochrane Central Register of Controlled Trials for all trials comparing intra-articular therapies was conducted on November 12, 2018. The treatments assessed were high molecular weight and low molecular weight hyaluronic acid injections, extended-release corticosteroids, standard-release corticosteroids, platelet-rich plasma, and saline. A frequentist network meta-analysis was conducted for each outcome.

**Results:**

Sixty-four articles (9710 patients) met the inclusion criteria. High molecular weight hyaluronic acid (− 0.53, 95% CI − 0.81 to − 0.25) and PRP (− 0.79, 95% CI − 1.32 to − 0.26) were the only treatments with a confidence interval that lay completely above the MID threshold; however, PRP results varied within sensitivity analyses. For the function analysis, high molecular weight hyaluronic acid (SMD − 0.76, 95% CI − 1.30 to − 0.22) was the only treatment with a confidence interval entirely above the MID. Extended-release corticosteroid demonstrated a possible benefit in functional improvement (SMD − 0.98, 95% CI − 1.79 to − 0.17) compared to that of standard-release corticosteroid (SMD − 0.14, 95% CI − 0.72 to 0.44).

**Conclusion:**

High molecular weight HA was the only treatment to surpass the MID for both pain and function outcomes. Extended-release corticosteroids may provide additional clinical benefit over standard-release corticosteroids. Platelet-rich plasma demonstrated possibly beneficial results; however, wide confidence intervals and sensitivity analyses made the conclusions of efficacy uncertain.

**Level of evidence:**

Level 1. Systematic review of level 1 evidence.

**Electronic supplementary material:**

The online version of this article (10.1007/s00167-019-05763-1) contains supplementary material, which is available to authorized users.

## Introduction

Knee osteoarthritis (OA) is a common disease associated with progressive deterioration of the cartilage and narrowing of the joint space. Previous estimates note that 27 million adults have knee OA in the United States; a number that is continually growing due to the aging population [11, 12]. Patients often advance through a variety of treatment stages as their disease progresses, ranging from conservative management and oral anti-inflammatories to eventual knee arthroplasty if the disease advances to more severe stages [12]. A large proportion of knee OA patients reside in the mild-to-moderate stages of knee OA, where non-surgical intervention is needed to provide pain relief and limit functional impairment [13]. These patients are often treated with intra-articular (IA) injectable drugs; most commonly corticosteroids or hyaluronic acid (HA), while platelet-rich plasma (PRP) injections have been more recently investigated as a potential knee OA treatment option [12]. Many randomized clinical trials (RCTs) and meta-analyses have been published comparing various knee OA treatments to determine the effectiveness and safety of these interventions [6]. Evidence has suggested an earlier onset of clinical benefit with corticosteroids; however, longer-lasting effects have been seen with IA-HA [3, 4]. PRP has shown evidence of effectiveness and safety in a small number of studies, yet previous meta-analyses have demonstrated large amounts of imprecision regarding the estimates of PRP’s true effects [15]. Recent evidence exploring differentiating factors within classes of therapies has demonstrated improved efficacy by molecular weight (MW), and delivery mechanisms, such as microsphere technology [8].

The importance of identifying opioid-sparing treatments for chronic conditions, like osteoarthritis, has become a clear focus in the medical community. Recent guidelines support a judicious practice to opioid prescribing and general focus on sparing opioids whenever possible [9]. Pre-operative use of opioids is the strongest predictor of prolonged opioid use after surgery, which could be reduced by the use of other pain-reducing therapies for knee OA such as these IA therapies [7]. The purpose of this study was to evaluate the efficacy and safety of intra-articular treatments of primary knee OA using a network meta-analysis (NMA) design. Specifically, we aimed to compare IA-HA (high and low molecular weight), IA corticosteroid (standard and extended-release), PRP, and IA saline on the outcomes of pain and function, as well as treatment-related adverse events in patients with knee OA. We hypothesize that effects of knee OA treatments will differ due to differences in treatment characteristics.

## Materials and methods

### Literature search

A literature search of MEDLINE (through OVID), EMBASE (through OVID), Cochrane Central Register of Controlled Trials (CENTRAL) for all relevant studies was conducted on November 12, 2018. Online Appendix 1 presents the search strategy used. Hand-searches of the reference lists of retrieved articles were performed in attempt to identify any relevant studies that may have been missed by the search strategy.

### Study selection

Studies were included if: (1) one or more of the following IA-treatments are evaluated: corticosteroids, hyaluronic acid (HA), platelet-rich plasma (PRP); (2) comparator was a placebo control or another eligible intervention; (3) primary or secondary outcome was pain measured at 3 ± 1 months, function measured at 3 ± 1 months, or treatment-related adverse effects; (4) the study was in English; (5) the study included only adults.

Using a standardized pilot-tested form, the eligibility assessment of the title and abstract of citations obtained from the search was performed by two independent reviewers (MP, AP, RP, MD, and ZG). All studies included by at least one of the reviewers in the title and abstract stage was screened in full text. Any disagreements at the full-text screening stage were resolved by consensus, and if consensus was not reached, a third reviewer was consulted.

### Data extraction

All data extraction was conducted using a standardized pilot-tested form. Data regarding study characteristics, patient demographics, treatments compared, and relevant outcomes was extracted. Any retrieved articles that were deemed to be reporting on the same patient population were included as a single study within the systematic review.

Data were extracted for pain, function, treatment-related adverse events. Pain and function outcomes were assessed at 3 months or closest follow-up time reported within ± 1 month. If repeated injection was conducted within the study, only the results following initial treatment were included. The following treatment groups were included within the network meta-analysis: High MW HA (≥ 3000 kDa), Low MW HA (< 3000 kDa), PRP, Standard-release corticosteroids, and Extended-release corticosteroids (Zilretta^®^, Flexion Therapeutics).

### Network geometry

A description of the network geometry, including the number of unique treatments and how frequently they are evaluated, as well as the comparisons between different treatments will be provided through a network plot. Network plots weighted nodes by the number of studies including the corresponding treatment and weighted connections by the number of studies comparing the two connected nodes.

### Measures of treatment effect

For continuous outcomes, standardized mean differences (SMD) with 95% confidence intervals were reported, as included trials used different instruments to assess pain and function. For treatment-related adverse events, risk ratios (RR) with 95% confidence intervals were calculated. All analyses were conducted using RStudio (RStudio Inc, Version 1.1.383) running R software (R Foundation, Version 3.5.0).

### Dealing with missing data

Mean changes from baseline values were estimated by subtracting the final mean from the baseline mean if a direct change was not reported. For the standard deviation (SD), an assumed within-group correlation of 0.5 was used to estimate the SD of the change from baseline between the groups. When insufficient information was available to calculate SDs for the change scores or when SDs at follow-up were not reported, the same SD as baseline was used. When no SDs were available, imputed SDs were used from other included trials of similar sample size and outcome measure. Intention-to-treat analysis was used whenever possible, unless it was not reported within the included study [10].

### Assessment of transitivity across treatment comparisons

To assess transitivity, study characteristics and pertinent patient demographics were assessed across each of the treatment comparison groups. The similarity of general details regarding the included patients across all treatment comparisons was assessed to ensure that the assumption of transitivity was met.

### Methods for direct and indirect or mixed treatment comparisons

Multiple NMAs were conducted to compare all included treatments simultaneously for each outcome. This study modelled the treatment contrast SMD for continuous outcomes and RR for dichotomous outcomes for two interventions as a function of comparisons between each individual intervention. The reference group was the saline control group. If a three-armed study reported on two interventions from the same group, the arm with the larger sample size was included. If the treatment arms were different doses of the same drug, the larger dose was used. The NMA was conducted using a frequentist random-effects model using the netmeta package in R (R Foundation, version 3.5.0).

Estimates of the overall ranks of treatments were conducted by calculating the *p* score for each treatment. The *p* score index will range between 0 and 100%, where the treatments with highest and lowest *p* scores are considered to be the best and worst treatments, respectively. Rankings will be provided for random-effects models for all outcomes. The rankings are based on effect estimates of each study relative to the Saline comparator.

Heterogeneity and inconsistency will be assessed for each outcome network using the *I*^2^ and Cochran’s *Q* statistic. Cochran’s *Q* and the corresponding *p* value will be reported for the model’s total heterogeneity/inconsistency, within-design heterogeneity, and between-design inconsistency. A heatmap was created for each outcome to identify comparisons within the network that contribute to the overall inconsistency.

### Sensitivity analysis

Sensitivity analyses were conducted to test the impact of any imputed data. These sensitivity analyses removed studies that had imputed data to ensure that these studies did not drastically impact the results of the network, as well as studies that were considered to be at high risk of bias based on the Cochrane’s risk of bias assessment of the allocation concealment domain.

## Results

### Study selection

**Fig. 1:**
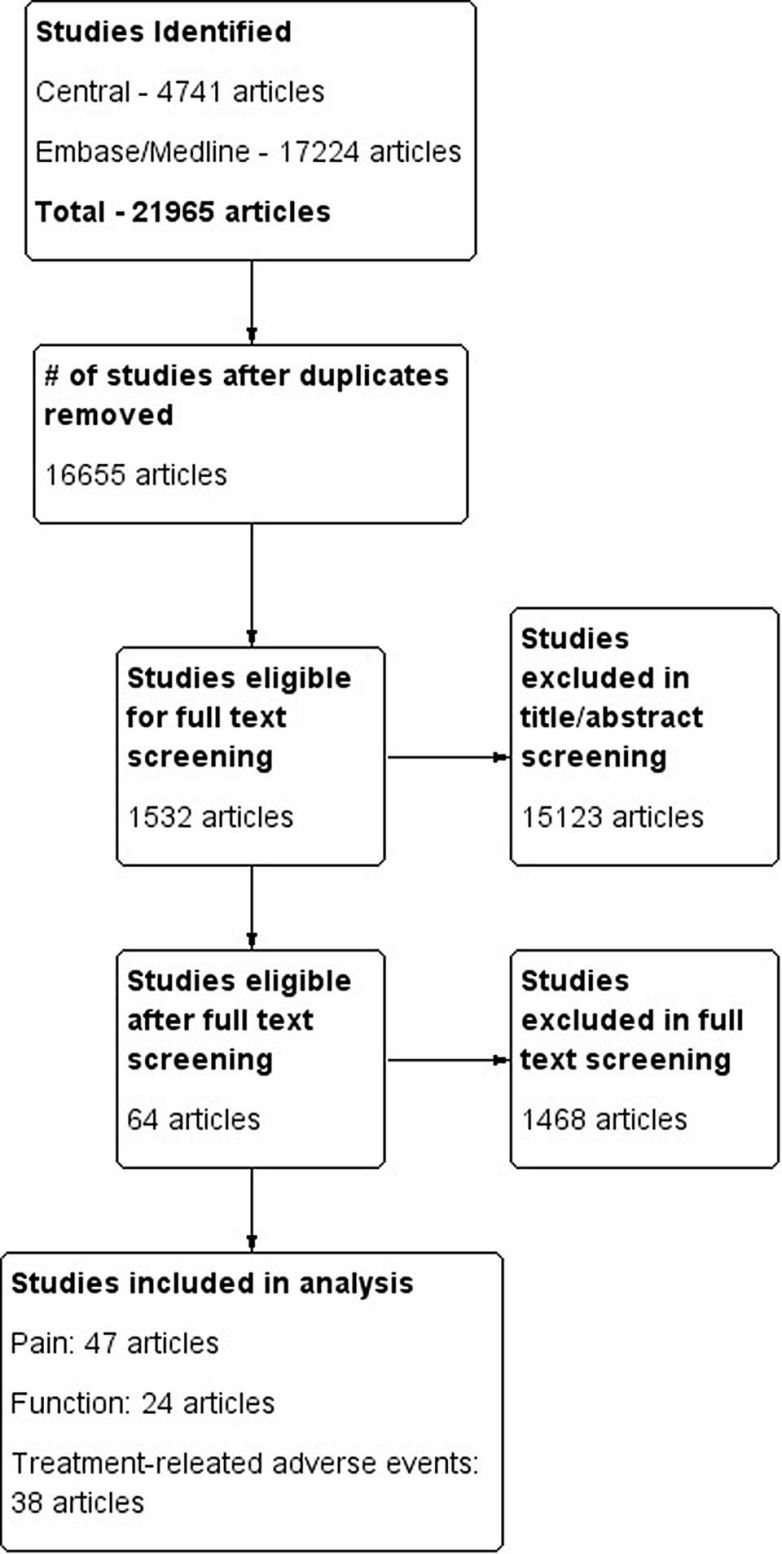
Study flow diagram

The conducted search identified a total of 21,965 articles on all available knee OA interventions (Fig. 1). After review of the full-text of these articles, a total of 64 articles met the inclusion criteria. Of these articles, 47 reported on pain at 3 months, 25 reported on function at 3 months, and 38 reported on treatment-related adverse events. A complete reference list of included studies is reported in Online Appendix 2. The Cochrane Risk of Bias assessment demonstrated that allocation sequence generation and allocation concealment were the most frequent categories in which studies may be at risk of bias, while blinding was typically adequate within many of the included studies. The entire Cochrane Risk of Bias assessment for each study can be found in Online Appendix 3.

### Pain

**Fig. 2:**
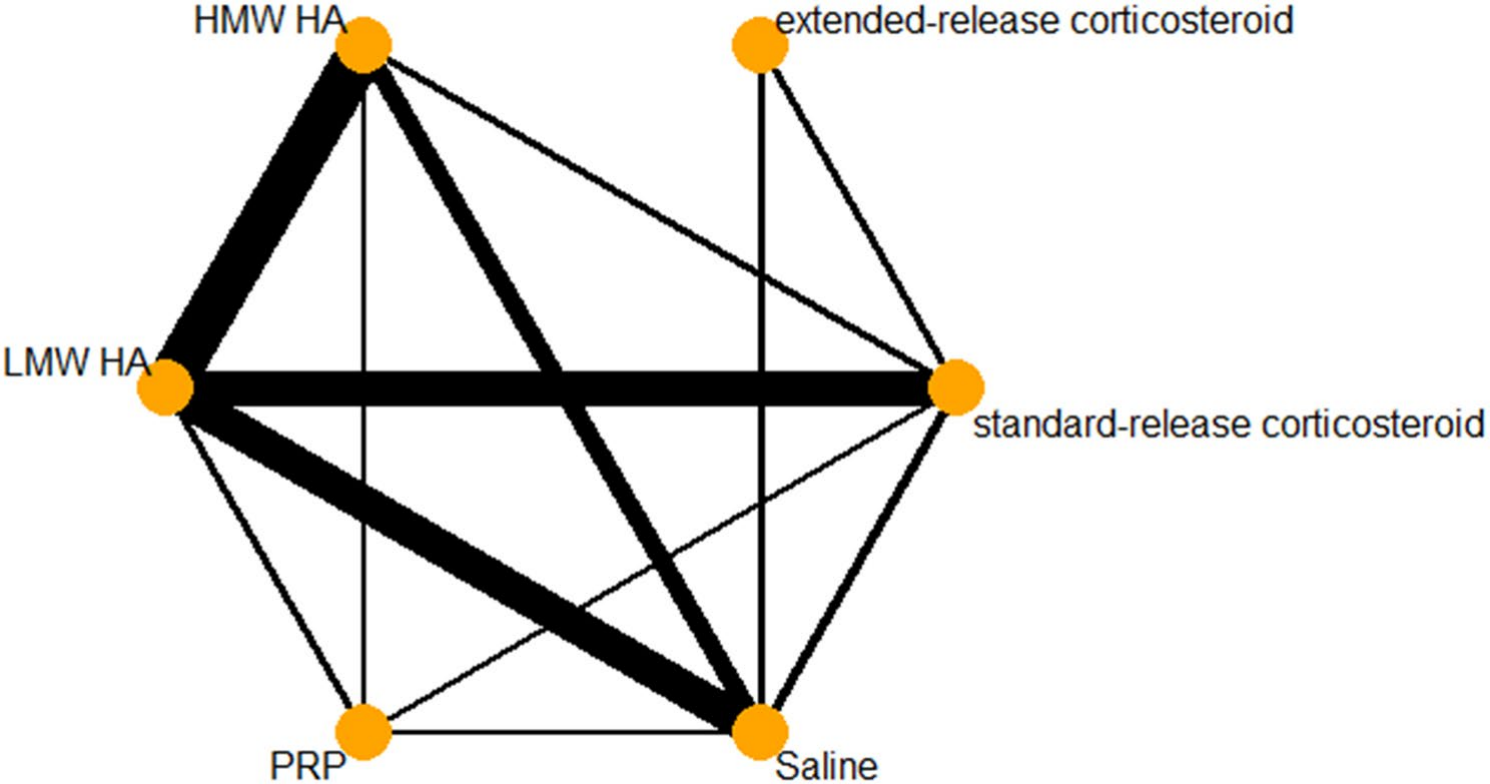
Pain network diagram

**Fig. 3:**
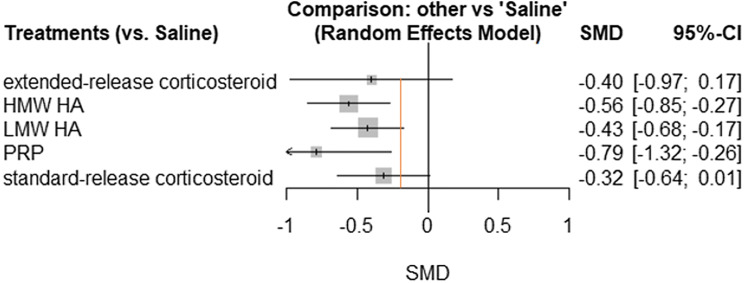
Pain forest plot. *Orange line indicates − 0.2 SD units, which is considered a small clinical benefit

There was a total of 53 pairwise comparisons within 47 trials included in the pain analysis. A network diagram for pain is provided in Fig. 2. A forest plot of each treatment compared to IA-saline for the outcome of pain is provided in Fig. 3. There were 19 trials including saline as a comparator, 20 assessing HMW HA, 33 assessing LMW HA, 5 assessing PRP, 3 assessing extended-release corticosteroid, and 17 assessing standard-release corticosteroids. Table 1 provides a summary of the treatments included within the network. When compared to a minimum important difference (MID) of − 0.2 SD units, HMW HA (− 0.56, 95% CI − 0.85 to − 0.27) and PRP (− 0.79, 95% CI − 1.32 to − 0.26) were the only treatments with a confidence interval that lay completely above the threshold. Extended-release corticosteroid, standard-release corticosteroid, LMW HA, and PRP all had point estimates above the -0.2 threshold; however, their confidence intervals crossed this threshold. The random-effects ranking based on point estimates ranked the treatments in the following order: PRP (#1: *p* score = 0.9070), HMW HA (#2: *p* score = 0.7366), LMW HA (#3: *p* score = 0.5063), extended-release corticosteroid (#4: *p* score = 0.4914), standard-release corticosteroid (#5: *p* score = 0.3358), and IA-saline (#6: *p* score = 0.0229).

**Table 1:** Descriptive statistics for included treatments in networks

Treatment	Number of studies	Number of patients	Products in treatment category	Included studies^a^
High MW HA—pain	22	2306	Synvisc Synvisc-one Euflexxa Gel-one Durolane	2, 4, 6, 7, 10, 13, 14, 25, 34, 35, 36, 41, 45, 46, 47, 48, 50, 56, 57, 64, 68
High MW HA—function	12	719		1, 3, 10, 11, 17, 35, 36, 37, 39, 41, 60, 65
High MW HA—adverse events	23	2563		1, 3, 4, 6, 10, 13, 25, 30, 34, 35, 36, 37, 39, 41, 42, 45, 48, 50, 56, 57, 64, 65, 68
Low MW HA—pain	35	2762	Hyalgan Supartz Orthovisc Monovisc Artzal	1, 5, 6, 9, 11, 18, 21, 22, 24, 25, 26, 29, 30, 33, 34, 35, 36, 38, 40, 41, 42, 43, 45, 46, 47, 50, 55, 57, 59, 60, 62, 64, 65, 68
Low MW HA—function	11	645		5, 6, 9, 21, 35, 36, 37, 38, 41, 54, 62
Low MW HA—adverse events	37	2996		1, 6, 7, 9, 18, 19, 20, 21, 24, 25, 27, 31, 32, 34, 35, 36, 37, 38, 40, 41, 43, 44, 45, 49, 50, 52, 53, 54, 57, 58, 60, 61, 62, 63, 64, 66, 68
Standard-release corticosteroid^b^—pain	18	1019	Triamcinolone Betamethasone Hydrocortisone Methylprednisolone Cortisone	5, 7, 8, 10, 11, 12, 16, 23, 24, 28, 30, 33, 40, 42, 59, 60, 62, 67
Standard-release corticosteroid^b^—function	10	628		5, 8, 10, 11, 15, 23, 54, 60, 62, 67
Standard-release corticosteroid^b^—adverse events	12	683		7, 8, 10, 24, 30, 40, 42, 51, 54, 60, 61, 62
Extended-release corticosteroid—pain	3	325	Zilretta	8, 15, 16
Extended-release corticosteroid—function	3	325		8, 15, 16
Extended-release corticosteroid—adverse events	3	325		8, 15, 16
PRP—pain	5	300	Platelet-rich plasma Autologous conditioned plasma	13, 20, 21, 22, 55
PRP—function	4	168		21, 23, 39, 55
PRP—adverse events	3	146		21, 38, 55

^a^Included study numbers are located within Online Appendix 2

^b^Any study that used an unspecified corticosteroid/glucocorticoid were considered to be standard-release corticosteroids

The pain network model demonstrated high heterogeneity and inconsistency. The *I*^2^ statistic for the model was 90.0%, with an overall Cochran’s *Q* of 449.31 (*p* < 0.0001). Tests for heterogeneity using Cochran’s *Q* determined a within-design *Q* value of 394.91 (*p* < 0.0001), while the test for between-design inconsistency found a *Q* value of 54.41 (*p* < 0.0001). A heatmap of inconsistency within the pain network is provided in Online Appendix 4. Sensitivity analysis to remove studies considered to have a high risk of bias due to improper allocation concealment demonstrated a decrease in the inconsistency metrics of the model, although they remained significant. Sensitivity analysis conducted to exclude studies with high risk of bias demonstrated changes to treatment estimates and rankings; particularly for the PRP data. The treatment SMD for PRP changed to − 0.30 (95% CI − 0.91 to 0.32). This resulted in a change in *p* score rankings, making HMW HA ranked #1 (*p* score = 0.8383), and PRP being ranked #2 (*p* score = 0.6397). Sensitivity analysis to remove studies with imputed standard deviations also impacted the clinical significance of PRP (− 0.67, 95% CI − 1.26 to − 0.08). Other treatment effects were minimally changed with the removal of imputed standard deviations.

### Function

**Fig. 4:**
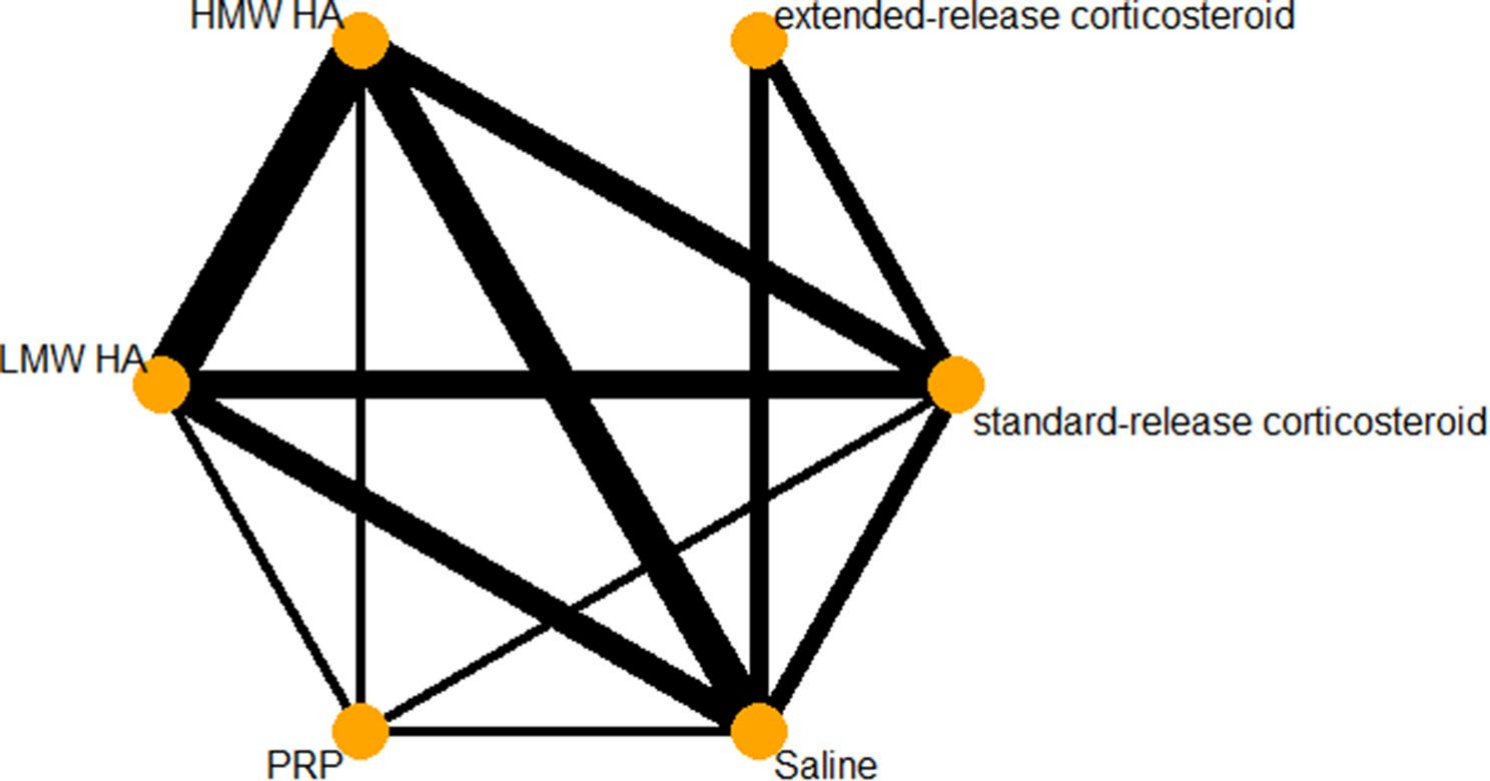
Function network diagram

**Fig. 5:**
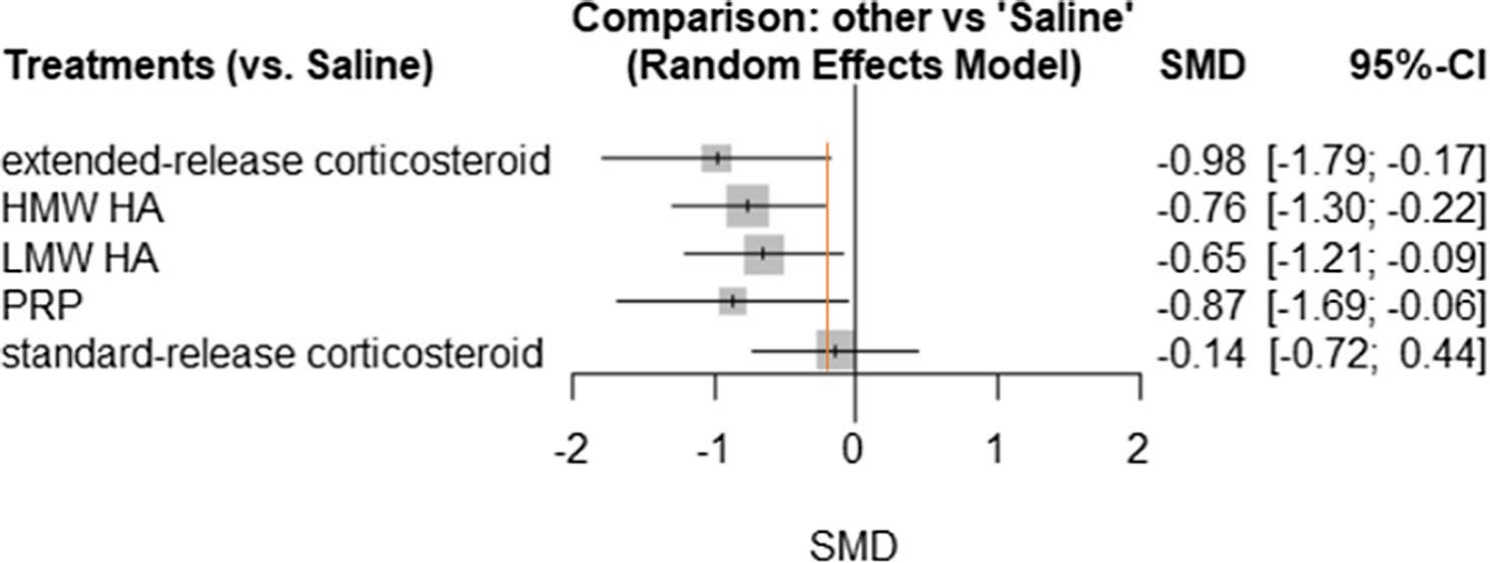
Function forest plot. *Orange line indicates − 0.2 SD units, which is considered a small clinical benefit

The network diagram for function is provided in Fig. 4. A total of 24 trials assessed function at the 3-month timepoint. HMW HA (SMD − 0.76, 95% CI − 1.30 to − 0.22) was the only treatment with a confidence interval entirely above the − 0.2 SD unit MID. Extended-release corticosteroids, LMW HA, and PRP all had point estimates above the MID threshold, however, their confidence intervals crossed over this threshold. Standard-release corticosteroids were the only treatment to have a point estimate below the − 0.2 cutoff, as well as have a confidence interval that crosses the line of no effect. A complete summary of network treatment effects in comparison to saline is provided in Fig. 5. The random-effects ranking based on point estimates ranked the treatments in the following order: extended-release corticosteroids (#1: *p* score = 0.8008), PRP (#2: *p* score = 0.7364), HMW HA (#3: *p* score = 0.6707), LMW HA (#4: *p* score = 0.0.5621), standard-release corticosteroids (#5: *p* score = 0.1583), and IA-Saline (#6: *p* score = 0.0716).

The function network demonstrated high heterogeneity and inconsistency. The model *I*^2^ was 92.8%, and total model Cochran’s *Q* was 292.32 (*p* < 0.0001). The within-design heterogeneity was significant (*Q* = 191.42, *p* < 0.0001), and between-design heterogeneity was also significant (*Q* = 100.90, *p* < 0.0001). A heatmap explaining inconsistency within the function network is provided in Online Appendix 4. Sensitivity analysis removing studies that were considered at high risk of improper allocation concealment demonstrated a reduction in the inconsistency within this analysis, although it was still significant (total model Cochran’s *Q* = 164.72, within-design *Q* = 79.49, between-design *Q* = 85.22). Sensitivity analysis for the function network meta-analysis was conducted by removing all studies that had imputed standard deviations, which had minimal impact on the results of the network.

### Treatment-related adverse events

**Fig. 6:**
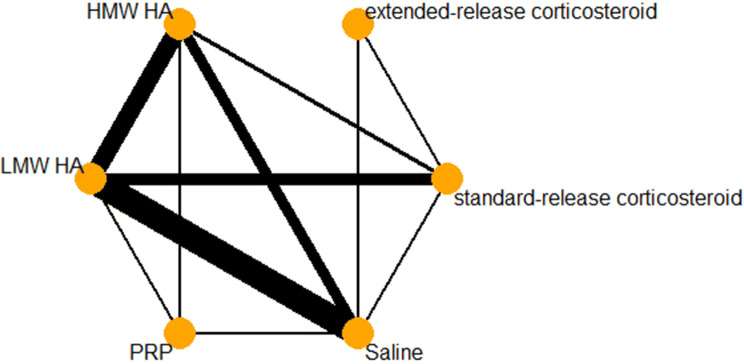
Adverse events network diagram

**Fig. 7:**
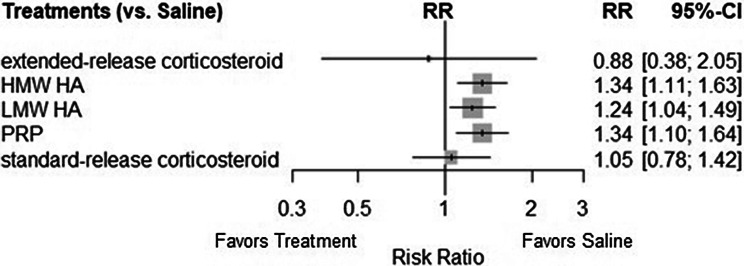
Treatment-related adverse event forest plot

There were 38 trials that assessed treatment-related adverse events across the 6 included treatments. A total of 40 direct pairwise comparisons were made within the network. The network diagram for treatment-related adverse events is provided in Fig. 6. The RRs of HMW HA (1.34, 95% CI 1.11–1.63), LMW HA (1.24, 95% CI 1.04–1.49), and PRP (1.34, 95% CI 1.10–1.64) all demonstrated a similar risk of treatment-related adverse events in relation to saline. These three treatments had confidence intervals that were completely on the side of an increased risk of treatment-related adverse event, while extended-release (0.88, 95% CI 0.38, 2.05) and standard-release corticosteroids (1.05, 95% CI 0.78, 1.42) both had confidence intervals that included the line of no difference. The confidence intervals of the corticosteroid groups largely overlapped the confidence intervals of HMW HA, LMW HA, and PRP. Risk ratios compared to saline are provided in Fig. 7 for all included treatments. There was no heterogeneity or inconsistency seen within the treatment-related adverse events (*I*^2^ = 0%, Cochran’s *Q* = 37.33, *p *= 0.8887).

## Discussion

The results of this study demonstrated consistent clinical improvements for both pain and function with the use of HMW HA in comparison to saline. LMW HA demonstrated slightly lower results, although confidence intervals did overlap with the HMW HA group. Extended-release corticosteroids demonstrated statistically significant and possible clinically significant results for function and pain. There may be clinical benefits to extended-release corticosteroids over standard-release corticosteroids; however, confidence interval overlap, and heterogeneity throughout the network precludes a definitive conclusion regarding superiority. The effect of PRP is currently unclear, particularly due to imprecision around the estimates of treatment effect and the aforementioned heterogeneity within the network, which stems from the limited number of studies and small sample sizes pooled for this therapy.

The results of this investigation comprehensively summarize the state of evidence for IA-injectables for knee OA by assessing the differentiating features between HA and corticosteroid products, while also including PRP therapy. The results for pain reduction, functional improvement, and treatment-related adverse events are similar to those seen in previous studies, while slight differences are seen due to the differentiation between products within drug classes [6]. Notably there is potential evidence of a prolonged treatment effect with extended-release corticosteroids in comparison to standard-release corticosteroids, particularly in the analysis of functional outcomes. While the extended-release corticosteroid estimates for pain reduction had wide confidence intervals, the estimates suggested a possible increase in effectiveness over standard corticosteroid counterparts. This could be further elucidated, or refuted, with additional future research that seeks to directly compare extended-release corticosteroids to both IA-saline, as well as standard-release corticosteroids. An important consideration when evaluating the results of this study is the potential treatment effect that has been demonstrated by IA-saline [2]. The evidence that IA-saline provides some therapeutic benefit suggests that the results presented in this study may be conservative estimates of the true effect of the assessed IA therapies due to the relative comparisons not being against a true null-effect comparator [1, 2].

HMW HA and LMW HA provided beneficial results regarding pain and function, yet the largest contributor to inconsistency and heterogeneity within the network was due to the IA-HA literature. There are many conflicting RCTs within the IA-HA literature regarding the clinical benefit in both pain and function improvements between HMW and LMW HA products, which results in inconsistency in the comparisons made throughout this NMA. An important consideration comes from the sensitivity analysis of studies that did not conceal allocation adequately. When removing these studies, the inconsistency between HMW and LMW HA evidence was reduced, although a significant amount of inconsistency remained. Similar to previous investigations, PRP has shown a potentially large treatment effect, yet there is a high amount of uncertainty around the estimates from the few trials available [15]. While extended-release corticosteroids and PRP rank high in this NMA, these rankings are limited by their lack of variance around the point estimates. This variance would be large based the forest plot comparisons of extended-release corticosteroids or PRP to IA-saline, making the NMA rankings of limited value in drawing conclusions. Instead, focus should be put on the treatment effect estimates and 95% confidence intervals seen within the forest plots for pain at three months.

The investigation of functional improvement demonstrated results for extended-release corticosteroids that held a possible clinically important improvement based on Cohen’s effect size cutoff. The rankings of treatments within the network suggested a distinct benefit of extended-release corticosteroids over the standard corticosteroid counterparts; however, the ranking system used in NMA analysis has serious aforementioned limitations. A previous NMA by Bannuru et al. provided improvement estimates within the range of these studies regarding corticosteroid effectiveness on function, although the current study provides an important differentiation within the corticosteroid class [6]. The standard-release corticosteroid estimate remains similar to that of Bannuru’s estimate for corticosteroids as a class, yet the estimate of extended-release corticosteroids in the current study is notably higher than that seen in the previous NMA [6]. This suggests that the differentiation between standard and extended-release corticosteroids may be an important consideration when deciding on a corticosteroid therapy, as not all products may demonstrate similar results at the three months timepoint.

In general, treatment-related adverse events were seen to be comparable across IA-HA and PRP products. These events were most commonly reported as minor acute flares that resolved themselves within days without further intervention. There is minimal evidence to suggest serious treatment-related adverse events are associated with any of the IA therapies investigated. Previous studies have also demonstrated an increased risk of minor adverse events, while showing general safety with regard to serious adverse events [5, 14].

A novel and important finding within this study is the potential differentiation between clinical outcomes of pain and function between extended-release and standard-release corticosteroids. Future investigations should aim to provide additional evidence on this comparison to reduce the uncertainty around the effect estimates of these therapies; thus, providing further clarity into the potential benefits that extended-release corticosteroids may provide. This finding, coupled with the clinical benefit of HMW IA-HA, provide findings that allow clinicians to better understand the importance of differentiating factors between products within the same class.

While the use of NMA methodology provides the ability to compare multiple available treatment options, there are still some limitations in the use of this methodology. The primary concern with current NMA analysis is the lack of precision provided by treatment rankings and the inconsistency of trial results. While the rankings may be robust in networks with low variance across included treatment effects, the current study included certain treatments with a high amount of variance in their effect estimates. Another limitation is the high inconsistency and heterogeneity seen within the IA therapy literature. Previous NMA analyses have also demonstrated this within their analyses, but this study aimed to provide a detailed insight into the causes of this inconsistency through novel heatmap generation. These heatmaps provide clear summaries of the comparisons within the network that are a cause of inconsistency. By conducting sensitivity analyses, this study also provided some plausible explanation for the causes of this inconsistency, particularly within the IA-HA literature.

## Conclusion

High molecular weight HA was the only treatment to surpass the MID for both pain and function outcomes. Extended-release corticosteroids may provide additional clinical benefit over standard-release corticosteroids. Platelet-rich plasma demonstrated possibly beneficial results; however, wide confidence intervals and sensitivity analyses made the conclusions of efficacy uncertain. Treatment-related adverse events may be less prevalent with the use of corticosteroids, but events seen in all therapies were primarily minor events that resolved on their own.

## Electronic supplementary material

Below is the link to the electronic supplementary material.

Supplementary file1 (DOCX 337 kb)
