# Supplementary material for: Differentiating factors of intra-articular injectables have a meaningful impact on knee osteoarthritis outcomes: a network meta-analysis
Source: Knee Surg Sports Traumatol Arthrosc. 2020 Jan 3;28(9):3031–9. doi: 10.1007/s00167-019-05763-1 (PMC7471203; doi:10.1007/s00167-019-05763-1)
Supplement: Supplementary file 1 — Supplementary file1 (DOCX 337 kb) [file 167_2019_5763_MOESM1_ESM.docx]

**Appendix 1**

| MEDLINE/EMBASE Search | CENTRAL Search |
| --- | --- |
| osteoarthritis/ OR degenerative disease/ | MeSH descriptor: [Osteoarthritis] this term only |
| (arthriti$ OR osteoarthriti$ OR degenerative OR arthros$ OR patell$ OR menisc$).tw. | arthriti* OR osteoarthriti* OR degenerative OR arthros* OR patell* OR menisc*:ti,ab,kw |
| or/1-2 | #1 OR #2 |
| knee joint/ OR patellofemoral joint/ | MeSH descriptor: [Knee Joint] this term only |
| knee/ OR patella/ | MeSH descriptor: [Knee] this term only |
| knee.tw. | knee:ti,ab,kw |
| or/4-6 | or/4-6 |
| 3 and 7 | #3 AND #7 |
| exp osteoarthritis, knee/ OR knee osteoarthritis/ OR knee arthritis/ OR knee disease/ | MeSH descriptor: [Osteoarthritis, Knee] explode all trees |
| gonarthros$.tw. | gonarthros*:ti,ab,kw |
| or/8-10 | (OR #8-#10) |
| glucocorticoids/ or Dexamethasone Isonicotinate/ or Dexamethasone/ or Prednisolone/ or Triamcinolone Acetonide/ or Triamcinolone/ or Betamethasone 17-Valerate/ or Betamethasone/ or Methylprednisolone Hemisuccinate/ or Methylprednisolone/ or Hydrocortisone/ or Adrenal Cortex Hormones/ | MeSH descriptor: [Glucocorticoids] this term only |
| (corticosteroid$ or steroid$ or prednisolone or cortivazol or triamcinolone or betamethasone or dexamethasone or hydrocortisone or glucocorticoid$ or hydroxycorticosteroid$ or corticoid$ or ketosteroid$ or androstenedione).tw. | MeSH descriptor: [Dexamethasone] this term only |
| viscosupplements/ OR viscosupplement/ | MeSH descriptor: [Dexamethasone Isonicotinate] this term only |
| hyaluronic acid/ | MeSH descriptor: [Prednisolone] this term only |
| (viscosupplement$ or hyaluronic acid or hyaluron$ or hylan$ or viskosup$ or synvisc$ or orthovisc$ or ostenil$ or suplasyn$ or arthrum$ or synov-hyal$ or artz$ or biotty$ or go-on$ or healon$ or hya-ject$ or hyalgan$ or hyalart$ or hyalectin$ or nuflexxa$ or polireumin$ or hy-gag$ or nrd101$ or replasyn$ or supartz$ or durolane$).tw. | MeSH descriptor: [Triamcinolone] this term only |
| (visco$ adj suppl$).tw. | MeSH descriptor: [Triamcinolone Acetonide] this term only |
| platelet-rich plasma/ OR thrombocyte rich plasma/ OR platelet-rich plasma cell/ | MeSH descriptor: [Betamethasone] this term only |
| platelet transfusion/ OR thrombocyte transfusion/ | MeSH descriptor: [Betamethasone Valerate] this term only |
| ("platelet-rich plasma" or "platelet rich plasma" or "PRP" or "platelet transfusion" or "plasma rich" or "autologous").tw. | MeSH descriptor: [Methylprednisolone] this term only |
| stem cells/ OR stem cell/ OR stem cell transplantation/ OR autologous stem cell transplantation/ | MeSH descriptor: [Methylprednisolone Hemisuccinate] this term only |
| mesenchymal stem cell transplantation/ | MeSH descriptor: [Hydrocortisone] this term only |
| mesenchymal stromal cell/ | MeSH descriptor: [Adrenal Cortex Hormones] this term only |
| (stem cell$ or mesenchym$ or bone marrow).tw. | corticosteroid* OR steroid* OR prednisolone OR cortivazol OR triamcinolone OR betamethasone OR dexamethasone OR hydrocortisone OR glucocorticoid* OR hydroxycorticosteroid* OR corticoid* OR ketosteroid* OR androstenedione:ti,ab,kw |
| or/12-24 | MeSH descriptor: [Viscosupplements] this term only |
| injections, intra-articular/ OR intraarticular drug administration/ | MeSH descriptor: [Hyaluronic Acid] this term only |
| (intraarticular inject$ or intra-articular inject$).tw. | viscosupplement* OR hyaluronic acid OR hyaluron* OR hylan* OR viskosup* OR synvisc* OR orthovisc* OR ostenil* OR suplasyn* OR arthrum* OR synov-hyal* OR artz* OR biotty* OR go-on* OR healon* OR hya-ject* OR hyalgan* OR hyalart* OR hyalectin* OR nuflexxa* OR polireumin* OR hy-gag* OR nrd101* OR replasyn* OR supartz* OR durolane*:ti,ab,kw |
| or/26-27 | visco* NEXT suppl*:ti,ab,kw |
| 25 and 28 | MeSH descriptor: [Platelet-Rich Plasma] this term only |
| orthotic devices/ OR orthosis/ | MeSH descriptor: [Platelet-Rich Fibrin] this term only |
| braces/ OR brace/ OR knee brace/ OR orthopedic equipment/ OR shoe/ | platelet-rich plasma OR platelet rich plasma OR PRP OR platelet transfusion OR plasma rich OR autologous:ti,ab,kw |
| (brace$ or bracing or orthotic$ or orthoses or orthosis or insole$ or sleeve$).tw. | MeSH descriptor: [Stem Cells] this term only |
| self care/ OR empowerment/ | MeSH descriptor: [Mesenchymal Stem Cell Transplantation] this term only |
| self management/ | MeSH descriptor: [Mesenchymal Stromal Cells] this term only |
| consumer participation/ | stem cell* OR mesenchym* OR bone marrow:ti,ab,kw |
| patient education/ OR education/ | (OR #12-#35) |
| “power (psychology)”/ | MeSH descriptor: [Injections, Intra-Articular] this term only |
| holistic health/ | intraarticular inject* OR intra-articular inject*:ti,ab,kw |
| social support/ | #37 OR #38 |
| adaptation, psychological/ | #36 AND #39 |
| psychotherapy/ | MeSH descriptor: [Orthotic Devices] this term only |
| behavior therapy/ OR behavior/ | MeSH descriptor: [Braces] this term only |
| mind-body therapies/ OR alternative medicine/ | MeSH descriptor: [Foot Orthoses] this term only |
| cognitive therapy/ | brace* OR bracing OR orthotic* OR orthoses OR orthosis OR insole* OR sleeve*:ti,ab,kw |
| psychoanalytic therapy/ OR psychoanalysis/ | MeSH descriptor: [Self Care] this term only |
| counseling/ | MeSH descriptor: [Self-Management] this term only |
| psychology, applied/ | MeSH descriptor: [Consumer Participation] this term only |
| ((self or symptom$) adj (care or help or manag$ or directed or monitor$ or efﬁcacy or admin$)).tw. | MeSH descriptor: [Patient Education as Topic] this term only |
| ((health or patient$) adj2 (educat$ or information)).tw. | MeSH descriptor: [Power (Psychology)] this term only |
| ((patient$ or consumer$) adj part$).tw. | MeSH descriptor: [Holistic Health] this term only |
| (empower$ or holistic or wholistic or cope or copes or coping).tw. | MeSH descriptor: [Social Support] this term only |
| (social adj (support or network$)).tw. | MeSH descriptor: [Adaptation, Psychological] this term only |
| (support adj system$).tw. | MeSH descriptor: [Psychotherapy] this term only |
| (psychotherap* or psychological intervention*).tw. | MeSH descriptor: [Behavior Therapy] this term only |
| (psycholog$ adj3 (intervention$ or therap$ or treatment$ or adjust$ or adapt$)).tw. | MeSH descriptor: [Mind-Body Therapies] this term only |
| (behav$ adj3 (therap$ or intervention$ or modification or adapt$)).tw. | MeSH descriptor: [Cognitive Therapy] this term only |
| ("activity scheduling" or "assertiveness training" or "aversion therap$" or "covert sensitization" or "behav$ contracting" or biofeedback or "contingency management" or "conversion therap$" or "distraction therap$" or "exposure therap$" or "abreaction therap$" or "systematic desensitization therap$" or "eye movement desensitization" reprocessing or EMDR or "implosive therap$" or "pleasant event$" or psychoeducation$ or "reciprocal inhibition therap$" or "relaxation technique$" or "autogenic training" or distraction$ or "response cost" or "guided imagery" or "sleep phase chronotherap$" or "social skills training" or "social effectiveness" or "cognitive behav$ therap$").tw. | MeSH descriptor: [Psychoanalytic Therapy] this term only |
| (cognitive adj3 therap*).tw. | MeSH descriptor: [Counseling] this term only |
| (CBT or "problem solving" or "rational emotive therap$" or "reality therap$" or reconstructuring or "role play" or schema$ or "self control" or "stress management" or "third wave therap$").tw. | MeSH descriptor: [Psychology, Applied] this term only |
| (acceptance adj3 commitment therap$).tw. | (self OR symptom*) NEXT (care OR help OR manag* OR directed OR monitor* OR efﬁcacy OR admin*):ti,ab,kw |
| (ACT or "behav$ activation" or compassion-focused or "dialectical behav$ therap$" or diffusion or "functional analytic psychotherap$" or "metacognitive therap$" or "mind training" or mindfulness).tw. | (health OR patient*) NEAR/2 (educat* OR information):ti,ab,kw |
| (psychodynamic adj3 psychotherap$).tw. | (patient* OR consumer*) NEXT part*:ti,ab,kw |
| (brief psychotherap$ or countertransference or freudian or "group therap$" or balint or jungian or klenian or "object relations" or "person centred therap$" or "client centred therap$" or "psychoanalytic therap$" or "alderian therap$" or "dream analysis" or "fee association" or "self analysis" or "short term psychotherap$" or transference or "humanistic therap$" or "existential therap$" or "experiential therap$" or "process experiential" or "gestalt therap$" or "expressive therap$" or "grief work$" or rogerian or "non directive therap$" or "supportive therap$" or "transactional analysis" or "integrative therap$" or "cognitive analytical therap$" or "eclectic therap$" or "interpersonal therap$" or multimodal or transtheoretical or "psychodynamic interpersonal therap$" or "systemic therap$" or "conjoint therap$" or "couples therap$" or "marital therap$" or "relationship therap$" or "emotion focused therap$" or "family therap$" or "integrative behavio?ral couple therap$" or "narrative therap$" or "personal construct" or "socioenvironmental therap$" or "solution focused brief therap$" or counsel$ or "directive counsel$" or "motivational interviewing").tw. | empower* OR holistic OR wholistic OR cope OR copes OR coping:ti,ab,kw |
| exercise/ OR aerobic exercise/ OR aquatic exercise/ OR dynamic exercise/ | social NEXT (support OR network*):ti,ab,kw |
| exertion/ | support NEXT system*:ti,ab,kw |
| physical fitness/ OR fitness/ | psychotherap* OR psychological intervention*:ti,ab,kw |
| exercise test/ | psycholog* NEAR/3 (intervention* OR therap* OR treatment* OR adjust* OR adapt*):ti,ab,kw |
| exercise tolerance/ | behav* NEAR/3 (therap* OR intervention* OR modification OR adapt*):ti,ab,kw |
| sport/ or sports/ | activity scheduling OR assertiveness training OR aversion therap* OR covert sensitization OR behav* contracting OR biofeedback OR contingency management OR conversion therap* OR distraction therap* OR exposure therap* OR abreaction therap* OR systematic desensitization therap* OR eye movement desensitization reprocessing OR EMDR OR implosive therap* OR pleasant event* OR psychoeducation* OR reciprocal inhibition therap* OR relaxation technique* OR autogenic training OR distraction* OR response cost OR guided imagery OR sleep phase chronotherap* OR social skills training OR social effectiveness OR cognitive behav* therap*:ti,ab,kw |
| pliability/ | cognitive NEAR/3 therap*:ti,ab,kw |
| physical endurance/ OR endurance/ | CBT OR problem solving OR rational emotive therap* OR reality therap* OR reconstructuring OR role play OR schema* OR self control OR stress management OR third wave therap*:ti,ab,kw |
| (exertion$ or exercis$ or sport$).tw. | acceptance NEAR/3 commitment therap*:ti,ab,kw |
| ((physical or motion) adj5 (ﬁtness or therap$)).tw. | ACT OR behav* activation OR compassion-focused OR dialectical behav* therap* OR diffusion OR functional analytic psychotherap* OR metacognitive therap* OR mind training OR mindfulness:ti,ab,kw |
| (physical$ adj2 endur$).tw. | psychodynamic NEAR/3 psychotherap*:ti,ab,kw |
| (strength$ or isometric$ or isotonic$ or isokinetic$ or aerobic$ or endurance or weight$) adj5 (exercis$ or train$)).tw. | brief psychotherap* OR countertransference OR freudian OR group therap* OR balint OR jungian OR klenian OR object relations OR person centred therap* OR client centred therap* OR psychoanalytic therap* OR alderian therap* OR dream analysis OR fee association OR self analysis OR short term psychotherap* OR transference OR humanistic therap* OR existential therap* OR experiential therap* OR process experiential OR gestalt therap* OR expressive therap* OR grief work* OR rogerian OR non directive therap* OR supportive therap* OR transactional analysis OR integrative therap* OR cognitive analytical therap* OR eclectic therap* OR interpersonal therap* OR multimodal OR transtheoretical OR psychodynamic interpersonal therap* OR systemic therap* OR conjoint therap* OR couples therap* OR marital therap* OR relationship therap* OR emotion focused therap* OR family therap* OR integrative behavio* couple therap* OR narrative therap* OR personal construct OR socioenvironmental therap* OR solution focused brief therap* OR counsel* OR directive counsel* OR motivational interviewing:ti,ab,kw |
| physical therapy modalities/ OR physiotherapy/ | MeSH descriptor: [Exercise] this term only |
| rehabilitation/ OR "exoskeleton (rehabilitation)"/ | MeSH descriptor: [Physical Exertion] this term only |
| (physiotherap$ or manipulat$ or kinesiotherap$ or "massage therap$" or "occupational therap$" or rehab$ or run$ or jog$ or treadmill$ or swim$ or balneotherapy or hydrotherap$ or "aquatic exercise$" or "aquatic sport$" or "pool therap$" or "water aerobic$" or "water exercise$" or "water run$" or "water training" or "water gymnastic$" or bicycl$ or cycle$ or cycling or walk$ or row$ or strength$).tw. | MeSH descriptor: [Physical Fitness] this term only |
| ("body weight" or "weight change$" or "weight loss" or "weight reduction" or "slimming" or "anti obesity" or "antiobesity" or "diet$").tw. | MeSH descriptor: [Exercise Test] this term only |
| bandages/ OR bandage/ | MeSH descriptor: [Exercise Tolerance] this term only |
| (tape$ or taping or bandag$).tw. | MeSH descriptor: [Sports] this term only |
| chondroitin/ | MeSH descriptor: [Pliability] this term only |
| chondroitin.tw. | MeSH descriptor: [Physical Endurance] this term only |
| acupuncture/ OR auricular acupuncture/ | exertion* OR exercis* OR sport*:ti,ab,kw |
| acupuncture therapy/ | (physical OR motion) NEAR/5 (ﬁtness OR therap*):ti,ab,kw |
| electroacupuncture/ | physical* NEAR/2 endur*:ti,ab,kw |
| (acupuncture or "acupuncture therap$" or auriculotherap$ or electroacupuncture).tw. | (strength* OR isometric* OR isotonic* OR isokinetic* OR aerobic* OR endurance OR weight*) NEAR/5 (exercis* OR train*):ti,ab,kw |
| ultrasonography/ OR echography/ | MeSH descriptor: [Physical Therapy Modalities] this term only |
| ultrasonic therapy/ OR ultrasound therapy/ | MeSH descriptor: [Rehabilitation] this term only |
| (ultrasound$ or ultrasonic$ or short wave therap$ or ultrasonograph$).tw. | physiotherap* OR manipulat* OR kinesiotherap* OR massage therap* OR occupational therap* OR rehab* OR run* OR jog* OR treadmill* OR swim* OR balneotherapy OR hydrotherap* OR aquatic exercise* OR aquatic sport* OR pool therap* OR water aerobic* OR water exercise* OR water run* OR water training OR water gymnastic* OR bicycl* OR cycle* OR cycling OR walk* OR row* OR strength*:ti,ab,kw |
| electric stimulation therapy/ OR electrotherapy/ OR muscle excitation/ | body weight OR weight change* OR weight loss OR weight reduction OR slimming OR anti obesity OR antiobesity OR diet*:ti,ab,kw |
| (electric$ adj (nerve or therap$ or stimulation or muscle)).tw. | MeSH descriptor: [Bandages] this term only |
| (electrostimulation or electroanalgesia or tens or altens or electroacupuncture or "neuromusc$ electric$" or "high volt" or pulsed or electromagnetic or electrotherap$ or iontophoresis or "transcutaneous nerve stimulation").tw. | MeSH descriptor: [Athletic Tape] this term only |
| cryotherapy/ | tape* OR taping OR bandag*:ti,ab,kw |
| temperature/ OR thermotherapy/ | MeSH descriptor: [Chondroitin] this term only |
| (cryo$ or cold$ or ice$ or icing or cool$ or heat or temperature$ or thermal$ or thermo$).tw. | chondroitin:ti,ab,kw |
| surgical Procedures, Operative/ OR surgery/ OR knee surgery/ | MeSH descriptor: [Acupuncture] this term only |
| debridement/ OR lavage/ | MeSH descriptor: [Acupuncture Therapy] this term only |
| arthroscopy/ OR arthroscopic surgery/ | MeSH descriptor: [Electroacupuncture] this term only |
| osteotomy/ | acupuncture OR acupuncture therap* OR auriculotherap*:ti,ab,kw |
| (surger$ or surgical or operat$).tw. | MeSH descriptor: [Ultrasonography] this term only |
| (debride$ or repair$).tw. | MeSH descriptor: [Ultrasonic Therapy] this term only |
| (resurfac$ or arthroscop$).tw. | ultrasound* OR ultrasonic* OR short wave therap* OR ultrasonograph*:ti,ab,kw |
| (needle$ adj4 debridement).tw. | MeSH descriptor: [Electric Stimulation Therapy] this term only |
| (needle$ adj4 irrigation).tw. | MeSH descriptor: [Transcutaneous Electric Nerve Stimulation] this term only |
| (needle$ adj4 lavage).tw. | electric* NEXT (nerve OR therap* OR stimulation OR muscle):ti,ab,kw |
| (needle$ adj4 washout).tw. | electrostimulation OR electroanalgesia OR tens OR altens OR electroacupuncture OR neuromusc* electric* OR high volt OR pulsed OR electromagnetic OR electrotherap* OR iontophoresis OR transcutaneous nerve stimulation:ti,ab,kw |
| joint adj4 lavage.tw. | MeSH descriptor: [Cryotherapy] this term only |
| (meniscectomy or chondroplast$ or osteotom$).tw. | MeSH descriptor: [Temperature] this term only |
| (device adj4 knee).tw. | cryo* OR cold* OR ice* OR icing OR cool* OR heat OR temperature* OR thermal* OR thermo*:ti,ab,kw |
| glucosamine/ OR n acetylglucosamine/ | MeSH descriptor: [Surgical Procedures, Operative] this term only |
| (glucosamine or acetylglucosamine or n-acetylglucosamine or n-acetyl-d-glucosamine).tw. | MeSH descriptor: [Debridement] this term only |
| anti-inﬂammatory Agents, non-steroidal/ OR nonsteroid antiinflammatory agent/ OR diclofenac/ OR diclofenac potassium/ OR naproxen/ OR ibuprofen/ OR celecoxib/ | MeSH descriptor: [Arthroscopy] this term only |
| acetaminophen/ OR paracetamol/ | MeSH descriptor: [Osteotomy] this term only |
| cyclooxygenase 2 inhibitors/ OR cyclooxygenase 2 inhibitor/ | surger* OR surgical OR operat*:ti,ab,kw |
| (nonsteroidal$ OR non-steroidal$ OR non steroidal$ OR nsaid$).tw. | debride* OR repair*:ti,ab,kw |
| (acetaminophen or hydroxyacetanilide or apap or n-acetyl-p-aminophenol or pacetamidophenol or p-hydroxyacetanilide or paracetamol or acetamidophenol or "n-4-hydroxyphenyl acetanilide" or acephen or acetaco or Tylenol or anacin-3 or "anacin 3" or anacin3 or datril or panadol or acamol or algotropyl).tw. | resurfac* OR arthroscop*:ti,ab,kw |
| (Diclophenac or Dicrofenac or Dichlofenal or "Diclofenac Sodium" or "Sodium Diclofenac" or Diclofenac, Sodium or "Diclonate P" or Feloran or Voltarol or Novapirina or Orthofen or Ortofen or Orthophen or SR-38 or SR 38 or SR38 or Voltaren or "Diclofenac Potassium" or GP-45,840 or "GP 45,840" or GP45,840 or DIEP or DHEP or Flector or "D-1-O-G cpd" or "D SG cpd").tw. | needle* NEAR/4 debridement:ti,ab,kw |
| (naproxen or napro$ or 22204-53-1 or Naprosyn or Naprosin or aleve or Synflex or proxen or anaprox or MNPA or Methoxypropiocin).tw. | needle* NEAR/4 irrigation:ti,ab,kw |
| ("alpha-Methyl-4-2-methylpropyl benzeneacetic Acid" or 15687-27-1 or Spedifen or Ibuprofen or N01ORX9D6S or Brufen or Ibumetin or Ibuprofen-Zinc or IP-82 or IP 82 or IP82 or Motrin or Nuprin or Rufen or Salprofen or "Trauma-Dolgit Gel" or "Trauma Dolgit Gel" or "TraumaDolgit Gel" or Saren or Arflamin or Flubenil or "AF 2259" or "MDC 917" or "NCX 4060" or pimeprofen).tw. | needle* NEAR/4 lavage:ti,ab,kw |
| (celecoxib or Celebrex or SC 58635 or SC-58635 or 169590-42-5 or 2,5-dimethyl-celecoxib or celebra or cobix or celcoxx or celexib or selecap).tw. | needle* NEAR/4 washout:ti,ab,kw |
| (cox 2 or cox-2).tw. | Joint NEAR/4 lavage:ti,ab,kw |
| Analgesics, Opioid/ OR opiate/ | meniscectomy OR chondroplast* OR osteotom*:ti,ab,kw |
| Narcotics/ OR narcotic analgesic agent/ | device NEAR/4 knee:ti,ab,kw |
| (acetyldihydrocodeine or alfentanil or allylprodine or alphamethylfentanyl or alphaprodine or benzylmorphine or betaprodine or bezitriamide or buprenorphine or nutorphanol or bremazocine or cartentan$ or codeine or contin or dextromoramide or dextropropoxyphene or dezocine or diacetylmorphine or diamorphine or dihydrocodeine or dihydromorphine or dihydromorphone or diphenoxylate or dipipanone or enadoline or ethylketazocine or ethylmorphine or etonitazene or etorphine or fentanyl or heroin or hydrocodone or hydromorphin$ or hydromorphone or ketazocine or ketobemidone or lefetamine or levomethadon or levomethadyl or levomethorphan or levorphanol or loperamide or meperidine or meptazinol or methadone or methadyl or methylmorphine or morphin$ or malbuphine or narcotic$ or nicocodeine or nicomorphine or normorphine or noscapin$ or ohmefentanyl or opiate$ or opioid$ or opium or oripavine or oxycodone or oxycontin or oxymorphone or papaveretum or papaverin or pentazocine or percocet or peronine or pethidine or phenazocine or phencyclidine or pholocodine or piritramid$ or prodine or promedol or propoxyphene or remifentanil or sufentanil or tapentadol or tramadol or thebaine or tilidine).tw. | MeSH descriptor: [Glucosamine] this term only |
| or/29-125 | glucosamine OR acetylglucosamine OR n-acetylglucosamine OR n-acetyl-d-glucosamine:ti,ab,kw |
| 11 AND 126 | MeSH descriptor: [Anti-Inﬂammatory Agents, Non-Steroidal] this term only |
| Limit 127 to ed=20120101-20180201 | MeSH descriptor: [Cyclooxygenase 2 Inhibitors] this term only |
| Limit 128 to dd=20120101-20180201 / Limit 128 to em=201201-201806 | MeSH descriptor: [Acetaminophen] this term only |
| arthroplasty/ | nonsteroidal* OR non-steroidal* OR non steroidal* OR nsaid*:ti,ab,kw |
| joint prosthesis/ | acetaminophen OR hydroxyacetanilide OR apap OR n-acetyl-p-aminophenol OR pacetamidophenol OR p-hydroxyacetanilide OR paracetamol OR acetamidophenol OR n-4-hydroxyphenyl acetanilide OR acephen OR acetaco OR Tylenol OR anacin-3 OR anacin 3 OR anacin3 OR datril OR panadol OR acamol OR algotropyl:ti,ab,kw |
| knee replacement/ or knee arthroplasty/ or total knee arthroplasty/ or total knee replacement/ | Diclophenac OR Dicrofenac OR Dichlofenal OR Diclofenac Sodium OR Sodium Diclofenac OR Diclofenac, Sodium OR Diclonate P OR Feloran OR Voltarol OR Novapirina OR Orthofen OR Ortofen OR Orthophen OR SR-38 OR SR 38 OR SR38 OR Voltaren OR Diclofenac Potassium OR GP-45,840 OR GP 45,840 OR GP45,840 OR DIEP OR DHEP OR Flector OR D-1-O-G cpd OR D SG cpd:ti,ab,kw |
| knee prosthesis/ | naproxen OR napro* OR 22204-53-1 OR Naprosyn OR Naprosin OR aleve OR Synflex OR proxen OR anaprox OR MNPA OR Methoxypropiocin:ti,ab,kw |
| "prostheses and implants"/ | alpha-Methyl-4-2-methylpropyl benzeneacetic Acid OR 15687-27-1 OR Spedifen OR Ibuprofen OR N01ORX9D6S OR Brufen OR Ibumetin OR Ibuprofen-Zinc OR IP-82 OR IP 82 OR IP82 OR Motrin OR Nuprin OR Rufen OR Salprofen OR Trauma-Dolgit Gel OR Trauma Dolgit Gel OR TraumaDolgit Gel OR Saren OR Arflamin OR Flubenil OR AF 2259 OR MDC 917 OR NCX 4060 OR pimeprofen:ti,ab,kw |
| arthroplasty, replacement, knee/ | celecoxib OR Celebrex OR SC 58635 OR SC-58635 OR 169590-42-5 OR 2,5-dimethyl-celecoxib OR celebra OR cobix OR celcoxx OR celexib OR selecap:ti,ab,kw |
| (arthroplast$ or hemiarthroplast$ or (joint$ adj2 replace$)).tw. | cox 2 OR cox-2 OR cyclooxygenase 2 inhibitors OR cyclooxygenase-2 inhibitors:ti,ab,kw |
| (surface$ adj replace$).tw. | MeSH descriptor: [Analgesics, Opioid] this term only |
| knee$.tw. AND (arthroplast$ or implant$ or replace$ or prosthe$ or endoprosthe$).tw. | MeSH descriptor: [Narcotics] this term only |
| capsaicin/ | acetyldihydrocodeine OR alfentanil OR allylprodine OR alphamethylfentanyl OR alphaprodine OR benzylmorphine OR betaprodine OR bezitriamide OR buprenorphine OR nutorphanol OR bremazocine OR cartentan* OR codeine OR contin OR dextromoramide OR dextropropoxyphene OR dezocine OR diacetylmorphine OR diamorphine OR dihydrocodeine OR dihydromorphine OR dihydromorphone OR diphenoxylate OR dipipanone OR enadoline OR ethylketazocine OR ethylmorphine OR etonitazene OR etorphine OR fentanyl OR heroin OR hydrocodone OR hydromorphin* OR hydromorphone OR ketazocine OR ketobemidone OR lefetamine OR levomethadon OR levomethadyl OR levomethorphan OR levorphanol OR loperamide OR meperidine OR meptazinol OR methadone OR methadyl OR methylmorphine OR morphin* OR malbuphine OR narcotic* OR nicocodeine OR nicomorphine OR normorphine OR noscapin* OR ohmefentanyl OR opiate* OR opioid* OR opium OR oripavine OR oxycodone OR oxycontin OR oxymorphone OR papaveretum OR papaverin OR pentazocine OR percocet OR peronine OR pethidine OR phenazocine OR phencyclidine OR pholocodine OR piritramid* OR prodine OR promedol OR propoxyphene OR remifentanil OR sufentanil OR tapentadol OR tramadol OR thebaine OR tilidine:ti,ab,kw |
| capsaicin derivative/ | (OR #40-#139) |
| capsaicin.tw. | #11 AND #140, January 2012 to February 2018, Limited to Trials |
| sodium salicylate/ OR salicylate sodium/ | MeSH descriptor: [Arthroplasty] this term only |
| salicylate.tw. | MeSH descriptor: [Joint Prosthesis] this term only |
| duloxetine/ OR duloxetine hydrochloride/ | MeSH descriptor: [Prostheses and Implants] this term only |
| duloxetine.tw. | MeSH descriptor: [Arthroplasty, Replacement, Knee] this term only |
| OR/130-145 | MeSH descriptor: [Knee Prosthesis] this term only |
| 11 AND 146 | arthroplast* OR hemiarthroplast* OR (joint* NEAR/2 replace*):ti,ab,kw |
| 129 OR 147 | surface* NEXT replace*:ti,ab,kw |
| randomized controlled trial.pt. | knee* AND (arthroplast* OR implant* OR replace* OR prosthe* OR endoprosthe*):ti,ab,kw |
| controlled clinical trial.pt. | MeSH descriptor: [Capsaicin] this term only |
| article.pt. | capsaicin:ti,ab,kw |
| randomi?ed.ti,ab. | MeSH descriptor: [Sodium Salicylate] this term only |
| placebo.ti,ab. | salicylate:ti,ab,kw |
| drug therapy.fs. | MeSH descriptor: [Duloxetine Hydrochloride] this term only |
| randomly.ti,ab. | duloxetine;ti,ab,kw |
| trial.ti,ab. | (OR #142-#155) |
| groups.ab. | #11 AND #156, Limit to Trials |
| or/149-157 | #141 OR #157 |
| 148 AND 158 |  |
| exp animals/ not humans/ |  |
| exp animal/ not human/ |  |
| 160 or 161 |  |
| 159 not 162 |  |

**Appendix 2**

**REFERENCE LIST OF INCLUDED STUDIES**

1. Altman, R. D., Akermark, C., Beaulieu, A. D., & Schnitzer, T. (2004). Efficacy and safety of a single intra-articular injection of non-animal stabilized hyaluronic acid (NASHA) in patients with osteoarthritis of the knee. Osteoarthritis Cartilage, 12(8), 642-649. doi:10.1016/j.joca.2004.04.010
2. Altman, R. D., & Moskowitz, R. (1998). Intraarticular sodium hyaluronate (Hyalgan) in the treatment of patients with osteoarthritis of the knee: a randomized clinical trial. Hyalgan Study Group. J Rheumatol, 25(11), 2203-2212.
3. Altman, R. D., Rosen, J. E., Bloch, D. A., Hatoum, H. T., & Korner, P. (2009). A double-blind, randomized, saline-controlled study of the efficacy and safety of EUFLEXXA for treatment of painful osteoarthritis of the knee, with an open-label safety extension (the FLEXX trial). Semin Arthritis Rheum, 39(1), 1-9. doi:10.1016/j.semarthrit.2009.04.001
4. Arden, N. K., Akermark, C., Andersson, M., Todman, M. G., & Altman, R. D. (2014). A randomized saline-controlled trial of NASHA hyaluronic acid for knee osteoarthritis. Curr Med Res Opin, 30(2), 279-286. doi:10.1185/03007995.2013.855631
5. Askari, A., Gholami, T., NaghiZadeh, M. M., Farjam, M., Kouhpayeh, S. A., & Shahabfard, Z. (2016). Hyaluronic acid compared with corticosteroid injections for the treatment of osteoarthritis of the knee: a randomized control trail. Springerplus, 5, 442. doi:10.1186/s40064-016-2020-0
6. Atamaz, F., Kirazli, Y., & Akkoc, Y. (2006). A comparison of two different intra-articular hyaluronan drugs and physical therapy in the management of knee osteoarthritis. Rheumatol Int, 26(10), 873-878. doi:10.1007/s00296-005-0096-x
7. Bisicchia, S., Bernardi, G., & Tudisco, C. (2016). HYADD 4 versus methylprednisolone acetate in symptomatic knee osteoarthritis: a single-centre single blind prospective randomised controlled clinical study with 1-year follow-up. Clin Exp Rheumatol, 34(5), 857-863.
8. Bodick, N., Lufkin, J., Willwerth, C., Kumar, A., Bolognese, J., Schoonmaker, C., . . . Clayman, M. (2015). An intra-articular, extended-release formulation of triamcinolone acetonide prolongs and amplifies analgesic effect in patients with osteoarthritis of the knee: a randomized clinical trial. J Bone Joint Surg Am, 97(11), 877-888. doi:10.2106/jbjs.n.00918
9. Brandt, K. D., Block, J. A., Michalski, J. P., Moreland, L. W., Caldwell, J. R., & Lavin, P. T. (2001). Efficacy and safety of intraarticular sodium hyaluronate in knee osteoarthritis. ORTHOVISC Study Group. Clin Orthop Relat Res(385), 130-143.
10. Caborn, D., Rush, J., Lanzer, W., Parenti, D., & Murray, C. (2004). A randomized, single-blind comparison of the efficacy and tolerability of hylan G-F 20 and triamcinolone hexacetonide in patients with osteoarthritis of the knee. J Rheumatol, 31(2), 333-343.
11. Campos, A. L. S., RSP, E. A., da Silva, E. B., Fayad, S. G., Acerbi, L. D., de Almeida, F. N., . . . Gameiro, V. S. (2017). Viscosupplementation in patients with severe osteoarthritis of the knee: six month follow-up of a randomized, double-blind clinical trial. Int Orthop, 41(11), 2273-2280. doi:10.1007/s00264-017-3625-9
12. Chao, J., Wu, C., Sun, B., Hose, M. K., Quan, A., Hughes, T. H., . . . Kalunian, K. C. (2010). Inflammatory characteristics on ultrasound predict poorer longterm response to intraarticular corticosteroid injections in knee osteoarthritis. Journal of Rheumatology, 37(3), 650-655.
13. Chevalier, X., Jerosch, J., Goupille, P., van Dijk, N., Luyten, F. P., Scott, D. L., . . . Pavelka, K. (2010). Single, intra-articular treatment with 6 ml hylan G-F 20 in patients with symptomatic primary osteoarthritis of the knee: a randomised, multicentre, double-blind, placebo controlled trial. Ann Rheum Dis, 69(1), 113-119. doi:10.1136/ard.2008.094623
14. Cole, B. J., Karas, V., Hussey, K., Pilz, K., & Fortier, L. A. (2017). Hyaluronic Acid Versus Platelet-Rich Plasma: A Prospective, Double-Blind Randomized Controlled Trial Comparing Clinical Outcomes and Effects on Intra-articular Biology for the Treatment of Knee Osteoarthritis. Am J Sports Med, 45(2), 339-346. doi:10.1177/0363546516665809
15. Conaghan, P. G., Cohen, S. B., Berenbaum, F., Lufkin, J., Johnson, J. R., & Bodick, N. (2017). Brief Report: A Phase IIb Trial of a Novel Extended-Release Microsphere Formulation of Triamcinolone Acetonide for Intraarticular Injection in Knee Osteoarthritis. Arthritis Rheumatol, 70(2), 204-211. doi:10.1002/art.40364
16. Conaghan, P. G., Hunter, D. J., Cohen, S. B., Kraus, V. B., Berenbaum, F., Lieberman, J. R., . . . Bodick, N. (2018). Effects of a Single Intra-Articular Injection of a Microsphere Formulation of Triamcinolone Acetonide on Knee Osteoarthritis Pain: A Double-Blinded, Randomized, Placebo-Controlled, Multinational Study. J Bone Joint Surg Am, 100(8), 666-677. doi:10.2106/jbjs.17.00154
17. Cubukcu, D., Ardic, F., Karabulut, N., & Topuz, O. (2005). Hylan G-F 20 efficacy on articular cartilage quality in patients with knee osteoarthritis: clinical and MRI assessment. Clin Rheumatol, 24(4), 336-341. doi:10.1007/s10067-004-1043-z
18. Day, R., Brooks, P., Conaghan, P. G., & Petersen, M. (2004). A double blind, randomized, multicenter, parallel group study of the effectiveness and tolerance of intraarticular hyaluronan in osteoarthritis of the knee. J Rheumatol, 31(4), 775-782.
19. Dixon, A. S., Jacoby, R. K., Berry, H., & Hamilton, E. B. (1988). Clinical trial of intra-articular injection of sodium hyaluronate in patients with osteoarthritis of the knee. Curr Med Res Opin, 11(4), 205-213. doi:10.1185/03007998809114237
20. Dougados, M., Nguyen, M., Listrat, V., & Amor, B. (1993). High molecular weight sodium hyaluronate (hyalectin) in osteoarthritis of the knee: a 1 year placebo-controlled trial. Osteoarthritis Cartilage, 1(2), 97-103.
21. Filardo, G., Di Matteo, B., Di Martino, A., Merli, M. L., Cenacchi, A., Fornasari, P., . . . Kon, E. (2015). Platelet-Rich Plasma Intra-articular Knee Injections Show No Superiority Versus Viscosupplementation: A Randomized Controlled Trial. Am J Sports Med, 43(7), 1575-1582. doi:10.1177/0363546515582027
22. Filardo, G., Kon, E., Di Martino, A., Di Matteo, B., Merli, M. L., Cenacchi, A., . . . Marcacci, M. (2012). Platelet-rich plasma vs hyaluronic acid to treat knee degenerative pathology: study design and preliminary results of a randomized controlled trial. BMC Musculoskelet Disord, 13, 229. doi:10.1186/1471-2474-13-229
23. Forogh, B., Mianehsaz, E., Shoaee, S., Ahadi, T., Raissi, G. R., & Sajadi, S. (2016). Effect of single injection of platelet-rich plasma in comparison with corticosteroid on knee osteoarthritis: a double-blind randomized clinical trial. J Sports Med Phys Fitness, 56(7-8), 901-908.
24. Frizziero, L., & Ronchetti, P. (2002). Intra-articular treatment of osteoarthritis of the knee: an arthroscopic and clinical comparison between sodium hyaluronate (500–730 kDa) and methylprednisolone acetate | SpringerLink. Journal of Orthoapedics and Traumatology, 3(2), 89-96. doi:10.1007/s101950200034
25. Gigis, I., Fotiadis, E., Nenopoulos, A., Tsitas, K., & Hatzokos, I. (2016). Comparison of two different molecular weight intra-articular injections of hyaluronic acid for the treatment of knee osteoarthritis. Hippokratia, 20(1), 26-31.
26. Hangody, L., Szody, R., Lukasik, P., Zgadzaj, W., Lenart, E., Dokoupilova, E., . . . Szendroi, M. (2018). Intraarticular Injection of a Cross-Linked Sodium Hyaluronate Combined with Triamcinolone Hexacetonide (Cingal) to Provide Symptomatic Relief of Osteoarthritis of the Knee: A Randomized, Double-Blind, Placebo-Controlled Multicenter Clinical Trial. Cartilage, 9(3), 276-283. doi:10.1177/1947603517703732]
27. Henderson EB, Smith EC, Pegley F, Blake DR (1994) Intra-articular injections of 750 kD hyaluronan in the treatment of osteoarthritis: a randomised single centre double-blind placebo-controlled trial of 91 patients demonstrating lack of efficacy. Ann Rheum Dis 53:529-534
28. Henriksen, M., Christensen, R., Klokker, L., Bartholdy, C., Bandak, E., Ellegaard, K., . . . Bliddal, H. (2015). Evaluation of the benefit of corticosteroid injection before exercise therapy in patients with osteoarthritis of the knee: a randomized clinical trial. JAMA Internal Medicine, 175(6), 923-930. doi:https://dx.doi.org/10.1001/jamainternmed.2015.0461
29. Henrotin, Y., Berenbaum, F., Chevalier, X., Marty, M., Richette, P., & Rannou, F. (2017). Reduction of the Serum Levels of a Specific Biomarker of Cartilage Degradation (Coll2-1) by Hyaluronic Acid (KARTILAGE CROSS) Compared to Placebo in Painful Knee Osteoarthritis Patients: The EPIKART Study, a Pilot Prospective Comparative Randomized Double Blind Trial. BMC Musculoskeletal Disorders, 18 (1). doi:http://dx.doi.org/10.1186/s12891-017-1585-2
30. Housman, L., Arden, N., Schnitzer, T. J., Birbara, C., Conrozier, T., Skrepnik, N., . . . Bailleul, F. (2014). Intra-articular hylastan versus steroid for knee osteoarthritis. Knee Surg Sports Traumatol Arthrosc, 22(7), 1684-1692. doi:10.1007/s00167-013-2438-7
31. Huang, T. L., Chang, C. C., Lee, C. H., Chen, S. C., Lai, C. H., & Tsai, C. L. (2011). Intra-articular injections of sodium hyaluronate (Hyalgan(R)) in osteoarthritis of the knee. a randomized, controlled, double-blind, multicenter trial in the Asian population. BMC Musculoskelet Disord, 12, 221. doi:10.1186/1471-2474-12-221
32. Huskisson EC, Donnelly S (1999) Hyaluronic acid in the treatment of osteoarthritis of the knee. Rheumatology (Oxford) 38:602-607
33. Jones, A. C., Pattrick, M., Doherty, S., & Doherty, M. (1995). Intra-articular hyaluronic acid compared to intra-articular triamcinolone hexacetonide in inflammatory knee osteoarthritis. Osteoarthritis Cartilage, 3(4), 269-273.
34. Juni, P., Reichenbach, S., Trelle, S., Tschannen, B., Wandel, S., Jordi, B., . . . Egger, M. (2007). Efficacy and safety of intraarticular hylan or hyaluronic acids for osteoarthritis of the knee: a randomized controlled trial. Arthritis Rheum, 56(11), 3610-3619. doi:10.1002/art.23026
35. Karatosun, V., Unver, B., Gocen, Z., & Sen, A. (2005). Comparison of two hyaluronan drugs in patients with advanced osteoarthritis of the knee. A prospective, randomized, double-blind study with long term follow-up. Clin Exp Rheumatol, 23(2), 213-218.
36. Karlsson, J., Sjogren, L. S., & Lohmander, L. S. (2002). Comparison of two hyaluronan drugs and placebo in patients with knee osteoarthritis. A controlled, randomized, double-blind, parallel-design multicentre study. Rheumatology (Oxford), 41(11), 1240-1248.
37. Khanasuk Y, Dechmaneenin T, Tanavalee A (2012) Prospective randomized trial comparing the efficacy of single 6-ml injection of hylan G-F 20 and hyaluronic acid for primary knee arthritis: a preliminary study. J Med Assoc Thai 95 Suppl 10:S92-97
38. Kul-Panza, E., & Berker, N. (2010). Is hyaluronate sodium effective in the management of knee osteoarthritis? A placebo-controlled double-blind study. Minerva Med, 101(2), 63-72.
39. Lana, J. F., Weglein, A., Sampson, S. E., Vicente, E. F., Huber, S. C., Souza, C. V., . . . Belangero, W. D. (2016). Randomized controlled trial comparing hyaluronic acid, platelet-rich plasma and the combination of both in the treatment of mild and moderate osteoarthritis of the knee. J Stem Cells Regen Med, 12(2), 69-78.
40. Leardini, G., Mattara, L., Franceschini, M., & Perbellini, A. (1991). Intra-articular treatment of knee osteoarthritis. A comparative study between hyaluronic acid and 6-methyl prednisolone acetate. Clin Exp Rheumatol, 9(4), 375-381.
41. Lee, P. B., Kim, Y. C., Lim, Y. J., Lee, C. J., Sim, W. S., Ha, C. W., . . . Lee, S. C. (2006). Comparison between high and low molecular weight hyaluronates in knee osteoarthritis patients: open-label, randomized, multicentre clinical trial. J Int Med Res, 34(1), 77-87. doi:10.1177/147323000603400110
42. Leopold, S. S., Redd, B. B., Warme, W. J., Wehrle, P. A., Pettis, P. D., & Shott, S. (2003). Corticosteroid compared with hyaluronic acid injections for the treatment of osteoarthritis of the knee. A prospective, randomized trial. J Bone Joint Surg Am, 85-a(7), 1197-1203.
43. Lundsgaard, C., Dufour, N., Fallentin, E., Winkel, P., & Gluud, C. (2008). Intra-articular sodium hyaluronate 2 mL versus physiological saline 20 mL versus physiological saline 2 mL for painful knee osteoarthritis: a randomized clinical trial. Scand J Rheumatol, 37(2), 142-150. doi:10.1080/03009740701813103
44. Navarro-Sarabia, F., Coronel, P., Collantes, E., Navarro, F. J., de la Serna, A. R., Naranjo, A., . . . Herrero-Beaumont, G. (2011). A 40-month multicentre, randomised placebo-controlled study to assess the efficacy and carry-over effect of repeated intra-articular injections of hyaluronic acid in knee osteoarthritis: the AMELIA project. Ann Rheum Dis, 70(11), 1957-1962. doi:10.1136/ard.2011.152017
45. Pavelka, K., & Uebelhart, D. (2011). Efficacy evaluation of highly purified intra-articular hyaluronic acid (Sinovial((R))) vs hylan G-F20 (Synvisc((R))) in the treatment of symptomatic knee osteoarthritis. A double-blind, controlled, randomized, parallel-group non-inferiority study. Osteoarthritis Cartilage, 19(11), 1294-1300. doi:10.1016/j.joca.2011.07.016
46. Petrella, R. J., Cogliano, A., & Decaria, J. (2008). Combining two hyaluronic acids in osteoarthritis of the knee: a randomized, double-blind, placebo-controlled trial. Clin Rheumatol, 27(8), 975-981. doi:10.1007/s10067-007-0834-4
47. Petrella, R. J., Emans, P. J., Alleyne, J., Dellaert, F., Gill, D. P., & Maroney, M. (2015). Safety and performance of Hydros and Hydros-TA for knee osteoarthritis: a prospective, multicenter, randomized, double-blind feasibility trial. BMC Musculoskelet Disord, 16, 57. doi:10.1186/s12891-015-0513-6
48. Petrella, R. J., & Petrella, M. (2006). A prospective, randomized, double-blind, placebo controlled study to evaluate the efficacy of intraarticular hyaluronic acid for osteoarthritis of the knee. J Rheumatol, 33(5), 951-956.
49. Pietrogrande, V., Melanotte, P. L., D'Agnolo, B., Ulivi, M., Benigni, G. A., Turchetto, L., ... Perbellini, A. (1991). Hyaluronic acid versus methylprednisolone intra-articularly injected for treatment of osteoarthritis of the knee. *Current Therapeutic Research*, *50*(5), 691-701.
50. Raman, R., Dutta, A., Day, N., Sharma, H. K., Shaw, C. J., & Johnson, G. V. (2008). Efficacy of Hylan G-F 20 and Sodium Hyaluronate in the treatment of osteoarthritis of the knee -- a prospective randomized clinical trial. Knee, 15(4), 318-324. doi:10.1016/j.knee.2008.02.012
51. Raynauld, J. P., Buckland-Wright, C., Ward, R., Choquette, D., Haraoui, B., Martel-Pelletier, J., . . . Pelletier, J. P. (2003). Safety and efficacy of long-term intraarticular steroid injections in osteoarthritis of the knee: a randomized, double-blind, placebo-controlled trial.[Erratum appears in Arthritis Rheum. 2003 Nov;48(11):3300]. Arthritis & Rheumatism, 48(2), 370-377.
52. Scale, D., Wobig, M., & Wolpert, W. (1994). Viscosupplementation of osteoarthritic knees with hylan: a treatment schedule study. Current Therapeutic Research, 55(3), 220-232.
53. Schwappach, J., Dryden, S. M., & Salottolo, K. M. (2017). Preliminary Trial of Intra-articular LMWF-5A for Osteoarthritis of the Knee. Orthopedics, 40(1), e49-e53. doi:10.3928/01477447-20160926-02
54. Skwara, A., Peterlein, C. D., Tibesku, C. O., Rosenbaum, D., & Fuchs-Winkelmann, S. (2009). Changes of gait patterns and muscle activity after intraarticular treatment of patients with osteoarthritis of the knee: a prospective, randomised, doubleblind study. Knee, 16(6), 466-472. doi:10.1016/j.knee.2009.03.003
55. Smith, P. A. (2016). Intra-articular Autologous Conditioned Plasma Injections Provide Safe and Efficacious Treatment for Knee Osteoarthritis: An FDA-Sanctioned, Randomized, Double-blind, Placebo-controlled Clinical Trial. American Journal of Sports Medicine, 44(4), 884-891. doi:https://dx.doi.org/10.1177/0363546515624678
56. Strand, V., Baraf, H. S. B., Lavin, P. T., Lim, S., & Hosokawa, H. (2012). A multicenter, randomized controlled trial comparing a single intra-articular injection of Gel-200, a new cross-linked formulation of hyaluronic acid, to phosphate buffered saline for treatment of osteoarthritis of the knee. Osteoarthritis and Cartilage, 20(5), 350-356.
57. Sun, S. F., Hsu, C. W., Lin, H. S., Liou, I. H., Chen, Y. H., & Hung, C. L. (2017). Comparison of Single Intra-Articular Injection of Novel Hyaluronan (HYA-JOINT Plus) with Synvisc-One for Knee Osteoarthritis: A Randomized, Controlled, Double-Blind Trial of Efficacy and Safety. J Bone Joint Surg Am, 99(6), 462-471. doi:10.2106/jbjs.16.00469
58. Tamir, E., Robinson, D., Koren, R., Agar, G., & Halperin, N. (2000). Intra-articular hyaluronan injections for the treatment of osteoarthritis of the knee: a randomized, double blind, placebo controlled study. Clinical and experimental rheumatology, 19(3), 265-270.
59. Tammachote, N., Kanitnate, S., Yakumpor, T., & Panichkul, P. (2016). Intra-Articular, Single-Shot Hylan G-F 20 Hyaluronic Acid Injection Compared with Corticosteroid in Knee Osteoarthritis: A Double-Blind, Randomized Controlled Trial. J Bone Joint Surg Am, 98(11), 885-892. doi:10.2106/jbjs.15.00544
60. Tasciotaoglu, F., & Oner, C. (2003). Efficacy of intra-articular sodium hyaluronate in the treatment of knee osteoarthritis. Clin Rheumatol, 22(2), 112-117. doi:10.1007/s10067-002-0690-1
61. Tekeoglu, I., Adak, B., Goeksoy, T., & Tosun, N. (1998). Effects of intra-articular injections of sodium hyaluronate (Orthovisc) and betamethasone on osteoarthritis of the knee. ROMATOLOJI VE TIBBI REHABILITASYON DERGISI.
62. Trueba Davalillo, C. A., Trueba Vasavilbaso, C., Navarrete Alvarez, J. M., Coronel Granado, P., Garcia Jimenez, O. A., Gimeno Del Sol, M., & Gil Orbezo, F. (2015). Clinical efficacy of intra-articular injections in knee osteoarthritis: a prospective randomized study comparing hyaluronic acid and betamethasone. Open Access Rheumatol, 7, 9-18. doi:10.2147/oarrr.s74553
63. van der Weegen, W., Wullems, J. A., Bos, E., Noten, H., & van Drumpt, R. A. (2015). No difference between intra-articular injection of hyaluronic acid and placebo for mild to moderate knee osteoarthritis: a randomized, controlled, double-blind trial. Journal of Arthroplasty, 30(5), 754-757. doi:https://dx.doi.org/10.1016/j.arth.2014.12.012
64. Wobig, M., Bach, G., Beks, P., Dickhut, A., Runzheimer, J., Schwieger, G., . . . Balazs, E. (1999). The role of elastoviscosity in the efficacy of viscosupplementation for osteoarthritis of the knee: a comparison of hylan G-F 20 and a lower-molecular-weight hyaluronan. Clin Ther, 21(9), 1549-1562.
65. Wobig, M., Dickhut, A., Maier, R., & Vetter, G. (1998). Viscosupplementation with hylan GF 20: a 26-week controlled trial of efficacy and safety in the osteoarthritic knee. Clinical therapeutics, 20(3), 410-423.
66. Wu, J.-J., Shih, L.-Y., Hsu, H.-C., & Chen, T.-H. (1997). The double-blind test of sodium hyaluronate (ARTZ) on osteoarthritis knee. Chinese medical journal-taipei-, 59, 99-106.
67. Yavuz, U., Sokucu, S., Albayrak, A., & Ozturk, K. (2012). Efficacy comparisons of the intraarticular steroidal agents in the patients with knee osteoarthritis. Rheumatology International, 32(11), 3391-3396.
68. Zhang, H., Zhang, K., Zhang, X., Zhu, Z., Yan, S., Sun, T., . . . Lin, J. (2015). Comparison of two hyaluronic acid formulations for safety and efficacy (CHASE) study in knee osteoarthritis: a multicenter, randomized, double-blind, 26-week non-inferiority trial comparing Durolane to Artz. Arthritis Res Ther, 17, 51. doi:10.1186/s13075-015-0557-x

**Appendix 2 – Cochrane Risk of Bias Assessment**

| **Study** | **Was the allocation sequence adequately generated?** | **Was allocation adequately concealed?** | **Were outcome assessors adequately blinded?** | **Were participants adequately blinded?** | **Was loss to follow-up reasonable?** | **Are reports of the study free of suggestion of selective outcome reporting?** |
| --- | --- | --- | --- | --- | --- | --- |
| **Altman 1998** | Uncertain | Uncertain | Yes | Yes | Yes | Yes |
| **Altman 2004** | Yes | Yes | Yes | Yes | Yes | Yes |
| **Altman 2009** | Yes | Uncertain | Yes | Yes | Yes | Yes |
| **Arden, 2014** | Uncertain | Uncertain | Yes | Yes | Uncertain | Uncertain |
| **Askari, 2016** | Yes | Yes | Yes | Yes | Yes | Yes |
| **Atamaz 2006** | Yes | Yes | No | No | Yes | Uncertain |
| **Bisicchia, 2016** | Yes | Uncertain | Yes | No | Yes | Yes |
| **Bodick, 2015** | Yes | Yes | Yes | Yes | Yes | Yes |
| **Brandt 2001** | Uncertain | Yes | Yes | Yes | No | Uncertain |
| **Caborn 2004** | Uncertain | Uncertain | Yes | No | Yes | Yes |
| **Campos 2017** | Yes | Yes | Yes | Yes | Uncertain | Yes |
| **Chao 2010** | Uncertain | Uncertain | Yes | Yes | Uncertain | Yes |
| **Chevalier 2010** | Yes | Yes | Yes | Yes | Yes | Yes |
| **Cole, 2017** | Yes | Yes | Yes | Yes | Yes | Yes |
| **Conaghan 2017** | Yes | Yes | Yes | Yes | Yes | Yes |
| **Conaghan, 2018** | Yes | Yes | Yes | Yes | Yes | Yes |
| **Cubukcu 2005** | Uncertain | Uncertain | Uncertain | Uncertain | Yes | Yes |
| **Day 2004** | Yes | Yes | Yes | Yes | Yes | Yes |
| **Dixon 1988** | Uncertain | Uncertain | Yes | Yes | Yes | Yes |
| **Dougados 1993** | Uncertain | Uncertain | Yes | Yes | Yes | Yes |
| **Filardo 2012** | Yes | Yes | Yes | Yes | Yes | Yes |
| **Filardo, 2015** | Yes | Yes | Yes | Yes | Yes | Yes |
| **Forogh, 2016** | Uncertain | Uncertain | Yes | Yes | Yes | Yes |
| **Frizziero 2002** | Yes | Uncertain | Yes | No | Yes | Yes |
| **Gigis, 2016** | Yes | Uncertain | Yes | Yes | Yes | Uncertain |
| **Hangody 2018** | Yes | Yes | Yes | Yes | Yes | Yes |
| **Henriksen, 2015** | Yes | Yes | Yes | Yes | Yes | Yes |
| **Henrotin, 2017** | Yes | Uncertain | Yes | Yes | Yes | Uncertain |
| **Housman, 2014** | Yes | Uncertain | Yes | Yes | Uncertain | Yes |
| **Huang 2011** | Uncertain | Uncertain | Uncertain | Yes | Yes | Yes |
| **Jones 1995** | Uncertain | Uncertain | Yes | Yes | Yes | Yes |
| **Juni 2007** | Yes | Yes | Yes | Yes | Yes | Yes |
| **Karatosun 2005** | Uncertain | Uncertain | Yes | Yes | No | Yes |
| **Karlsson 2002** | Yes | Uncertain | Yes | Yes | Yes | Yes |
| **Kul-Panza 2010** | Yes | Uncertain | Yes | Yes | Yes | Yes |
| **Lana, 2016** | Uncertain | Yes | Yes | Yes | Yes | Uncertain |
| **Leardini 1991** | Uncertain | Uncertain | No | No | Yes | Uncertain |
| **Lee 2006** | Yes | Uncertain | Yes | No | Yes | Uncertain |
| **Leopold 2003** | Yes | Yes | Yes | No | Uncertain | Uncertain |
| **Lundsgaard 2008** | Yes | Yes | Yes | Yes | Yes | Uncertain |
| **Navarro-Sarabia 2011** | Yes | Yes | Yes | Yes | Uncertain | Yes |
| **Pavelka 2011** | Yes | Yes | Yes | Yes | Yes | Uncertain |
| **Petrella 2006** | Uncertain | Uncertain | Yes | Yes | Yes | Yes |
| **Petrella 2008** | Yes | Yes | Yes | Yes | Yes | Yes |
| **Petrella, 2015** | Yes | Yes | Yes | Yes | Yes | Yes |
| **Raman 2008** | Yes | Yes | Yes | Yes | Yes | Uncertain |
| **Raynauld 2003** | Uncertain | Uncertain | Yes | Yes | Yes | Yes |
| **Scale 1994** | Uncertain | Uncertain | Yes | Yes | Yes | Yes |
| **Schwappach 2017** | Uncertain | Uncertain | Yes | Yes | Yes | Yes |
| **Skwara 2009** | Yes | Yes | Yes | Yes | Uncertain | Yes |
| **Smith, 2016** | Yes | Uncertain | Yes | Yes | Yes | Yes |
| **Strand 2012** | Yes | Uncertain | Yes | Yes | Yes | Yes |
| **Sun, 2017** | Uncertain | Uncertain | Yes | Yes | Yes | Yes |
| **Tamir 2001** | Uncertain | Uncertain | Uncertain | Yes | Yes | Uncertain |
| **Tammachote, 2016** | Yes | Yes | Yes | Yes | Yes | Yes |
| **Tascioglu 2003** | Uncertain | Uncertain | Yes | No | Yes | Yes |
| **Tekeoglu 1998** | Uncertain | Uncertain | No | No | Yes | Yes |
| **Trueba Davalillo 2015** | Yes | Yes | Yes | Yes | Yes | Yes |
| **van der Weegen 2015** | Yes | Yes | Yes | Yes | Yes | Yes |
| **Wobig 1998** | Uncertain | Uncertain | Yes | Yes | Yes | Yes |
| **Wobig 1999** | Uncertain | Uncertain | Yes | Yes | Yes | Yes |
| **Wu 1997** | Uncertain | Uncertain | Uncertain | Yes | Yes | Yes |
| **Yavuz 2012** | Uncertain | Uncertain | No | Yes | Uncertain | Uncertain |
| **Zhang, 2015** | Yes | Yes | Yes | Yes | Yes | Yes |

**Appendix 4**

**Heatmap of inconsistency: Pain**


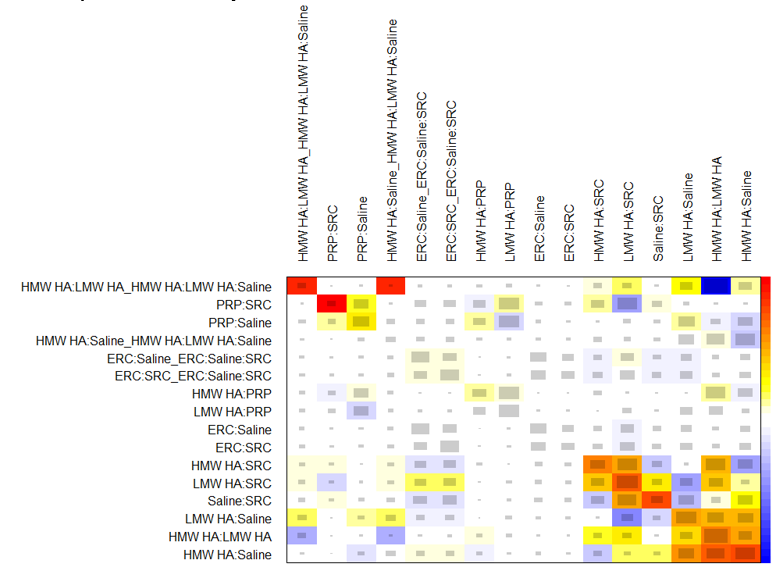


*ERC: Extended-release corticosteroid, SRC: Standard-release corticosteroid*

**Heatmap of inconsistency: Function**


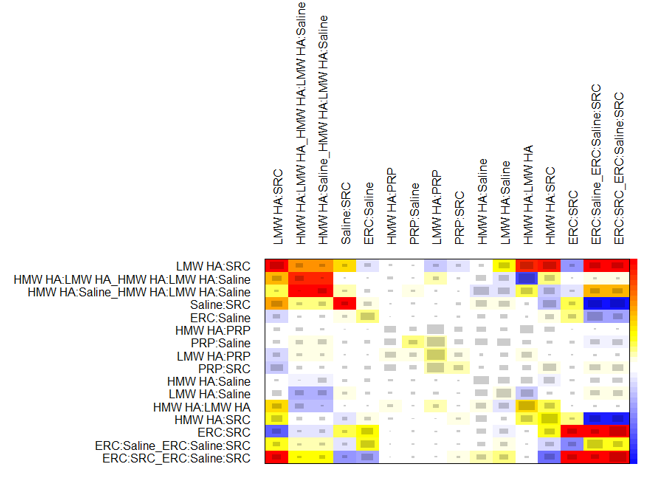


*ERC: Extended-release corticosteroid, SRC: Standard-release corticosteroid*

**Heatmap of Inconsistency: Adverse Events**


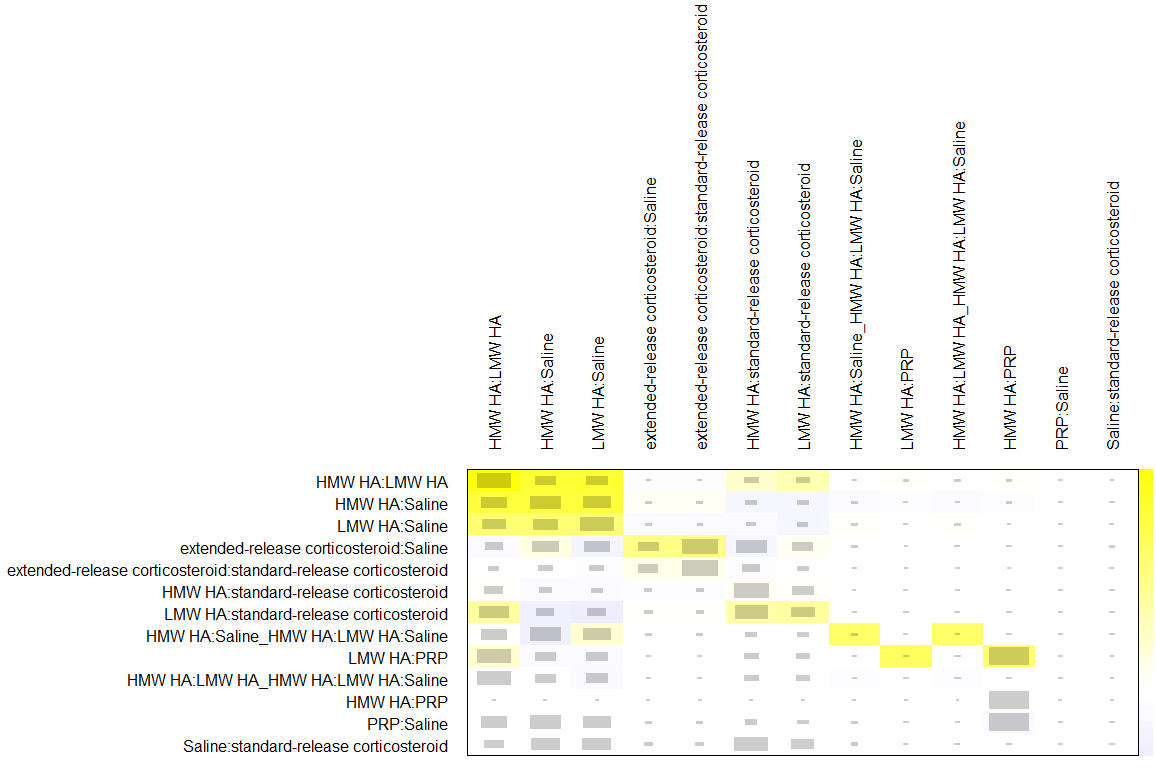


*ERC: Extended-release corticosteroid, SRC: Standard-release corticosteroid*
